# Experimental and Theoretical Studies of Isomeric Metal (N^C^N)Cl Coordination Complexes (Metal = Pt, Pd) with Multiple Conductance Pathways in Single-Molecule Junctions

**DOI:** 10.1021/acs.jpcc.5c07119

**Published:** 2026-02-06

**Authors:** Pablo Bastante, Ross J. Davidson, Yahia Chelli, Abdalghani H. S. Daaoub, Pilar Cea, Santiago Martin, Andrei S. Batsanov, Sara Sangtarash, Hatef Sadeghi, Martin R. Bryce, Nicolas Agrait

**Affiliations:** † Departamento de Física de la Materia Condensada C−III, and Instituto Universitario de Ciencia de Materiales “Nicolás Cabrera”, 16722 Universidad Autónoma de Madrid, Madrid E-28049, Spain; ‡ Department of Chemistry, 3057 Durham University, Durham DH1 3LE, U.K.; § Quantum Device Modelling Group, School of Engineering, 2707 University of Warwick, Coventry CV4 7AL, U.K.; ∥ Instituto de Nanociencia y Materiales de Aragón (INMA), 16765 CSIC-Universidad de Zaragoza, Zaragoza 50009, Spain; ⊥ Departamento de Química Física, Universidad de Zaragoza, Zaragoza 50009, Spain; # Laboratorio de Microscopias Avanzadas (LMA), Universidad de Zaragoza, Zaragoza 50018, Spain

## Abstract

The present work provides insight into the effect of connectivity within isomeric 3,5-bis­(pyridin-2-yl)­phenyl (N^C^N) platinum and palladium complexes on their electron transmission properties within gold|molecule|gold junctions. The ligands 3,5-bis­(4-(methylthio)­pyridin-2-yl)­phenyl hexanoate (**L** ^**m**^H) and 3,5-bis­(5-(methylthio)­pyridin-2-yl)­phenyl hexanoate (**L** ^**p**^H) were synthesized and coordinated with either PtCl or PdCl to form complexes **Pt** ^**m**^, **Pt** ^**p**^, **Pd** ^**m**^ and **Pd** ^**p**^. X-ray photoelectron spectroscopy (XPS) measurements evaluated the contacting modes of the molecules in the junctions. A combination of scanning tunneling microscopy-break junction (STM-BJ) measurements and density functional theory (DFT) calculations demonstrate that for the single-molecule S···S contacted junctions metal coordination enhanced the conductance compared with the free ligands. Notably, the higher degree of orbital mixing between the metal center and the ligand π-orbitals in the metal complexes plays a greater role than quantum interference to the extent that the complexes that incorporate ligands substituted with thiomethyl groups in *meta* positions relative to the pyridine-benzene linkages have a higher conductance than their *para*-analogs, e.g., **Pt** ^**p**^ −3.8 log­(*G*/*G*_0_) and **Pt** ^**m**^ −3.3 log­(*G*/*G*_0_), in contrast to the usual conductance trend (*para* > *meta*) for purely organic π-electron systems.

## Graphical abstract

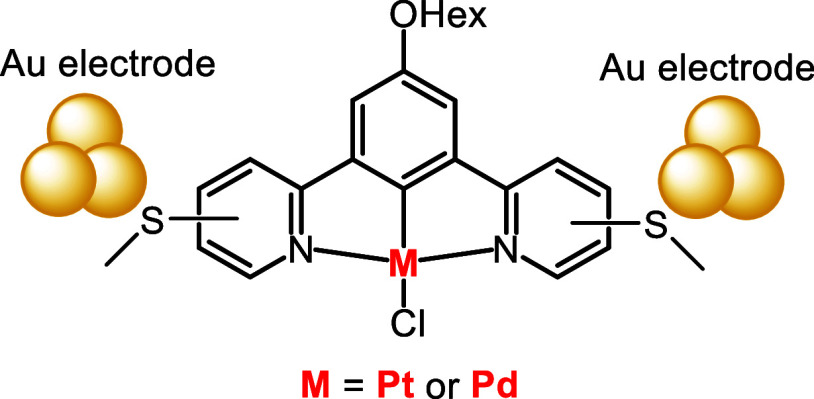

## Introduction

The fabrication of single-molecule junctions in which a molecule bridges two electrodes and the subsequent measurement of charge transport properties has enabled structure–property relationships in single-molecule conductance for a wide range of molecular wires, providing a large base for the design of future systems.^1^ Pure organic molecules [e.g., oligo­(phenyleneethynylenes)][Bibr ref2] have been extensively studied in this context.^3^ However, organometallic molecular wires are more scarce;^4^ they are of particular interest because the inclusion of single (or multiple) metal atom(s) into the molecule can impart rich structural and electronic properties such as diverse bonding patterns, a contraction of the energy gap between the highest occupied and lowest unoccupied molecular orbitals (the HOMO–LUMO gap) or more complex features such as redox activity or Kondo resonances.^5–8^

Most of the metal-containing architectures reported thus far can be divided into two broad categories: (i) metal–organic coordination complexes, often incorporating an imine-type nitrogen coordinated to the metal ion (e.g., 2,2′;6′,2″-terpyridine)^9^ or metalloporphyrins,^10^ and (ii) organometallic complexes containing a metal–carbon bond, e.g., metal bis­(acetylides) or metallocenes.^11–14^ Coordination complexes typically have poor orbital overlap between the d and π orbitals, resulting in orbitals with metal ion character being localized to the metal center, providing a means of introducing metal-specific features (e.g., spin crossover);^15^ however, such localization tends to result in low conductance, while the organometallic complexes often have greater orbital mixing, resulting in metal-containing orbitals being delocalized, leading to higher conductance.

Here, we examine a system that fits both architectures. The molecules contain both metal–nitrogen and metal–carbon bonding in the potential conductance paths, namely metal­(di­(2-pyridyl)­benzene)­Cl complexes [M­(N^C^N)­Cl] where M = Pt or Pd. Platinum­(II) complexes of N^C^N ligands [Pt­(N^C^N)­Cl] are of particular interest as phosphorescent emitters;^16^ they have high photoluminescent quantum yields and predictable structure–property relations, i.e., any modification made to either the metal center or the phenylate group alters the HOMO, while any change made to the pyridyl groups affects the LUMO. Additionally, owing to the square-planar geometry of the platinum in the Pt­(N^C^N)Cl complexes, they tend to undergo aggregation via weak metallophilic Pt···Pt contacts in addition to typical aromatic π–π stacking. The Pt···Pt interactions arise from effective d_*z*_^2^ and p_*z*_ orbital overlap of the metal centers,^17,18^ which results in the HOMO–LUMO energy gap being significantly smaller than that of the monomeric complex. Jones et al. proposed that such orbital overlaps would be capable of forming efficient conductive paths in a molecular junction.^19^ In a combined experimental and theoretical study we now unravel the different junction configurations and conductance pathways that operate in a series of four [M­(N^C^N)­Cl] complexes (M = Pt or Pd) in gold|molecule|gold junctions.

## Results and Discussion

### Molecular Design, Synthesis and Characterization

Two new isomeric N^C^N-based ligands were prepared to examine the conductance behavior of this class of organometallic complexes. The ligands were substituted with thiomethyl groups in either the *para* or *meta* positions relative to the pyridine-benzene bonds to provide anchoring groups to facilitate Au | molecule | Au junction formation.^20^ *n*-Hexanoate chains were attached to provide good solubility in organic solvents without sterically hindering subsequent metal complexation or junction formation, affording 3,5-bis­(4-(methylthio)­pyridin-2-yl)­phenyl hexanoate (**L** ^**m**^H) and 3,5-bis­(5-(methylthio)­pyridin-2-yl)­phenyl hexanoate (**L** ^**p**^H). The positional isomers of the terminal anchor groups were chosen to influence the potential multiple conductance paths in their metal complexes via py–M–py or py–(M–benzene)–py (M = Pt or Pd). While there are many examples of multiple conductance pathways in purely organic molecules which can adopt different binding configurations in the junction,^21–26^ studies on organometallic molecules are rare in this regard.^27,28^ Especially, the extent to which metal coordination can offer an additional charge transport pathway compared to the free ligand is not well understood.^29–32^ This topic has inspired the present molecular design.

Platinum­(II) and palladium­(II) complexes were synthesized using Cárdenas’ approach of heating the ligand with K_2_MCl_4_ (where M = Pt or Pd) in acetic acid^33^ to give the corresponding metal complexes **Pt** ^**m**^, **Pd** ^**m**^, **Pt** ^**p**^ or **Pd** ^**p**^ in 35–63% yields (Figure 1). The structures and high level of purity of the ligands and complexes were unambiguously established by NMR spectroscopy, mass spectrometry and elemental analysis (see Supporting Information). Absorption and emission spectra and additional photophysical data of the complexes are reported in the Supporting Information.

**Figure 1:**
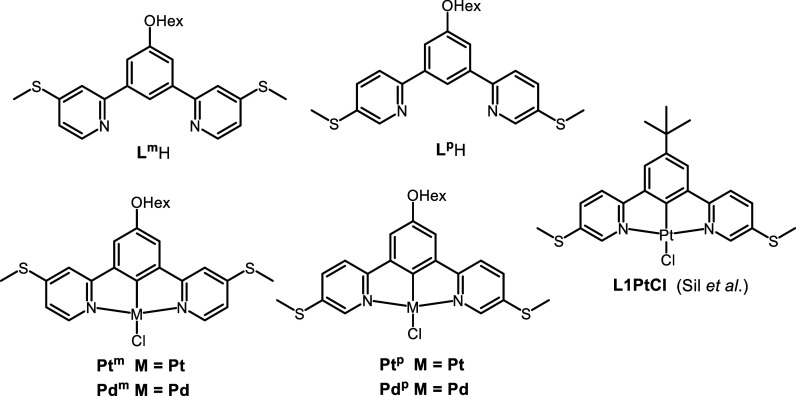
Structures of **L** ^**m**^H, **L** ^**p**^H, **Pt** ^**m**^, **Pd** ^**m**^, **Pt** ^**p**^, and **Pd** ^**p**^ where Hex = *n*-C_6_H_13_, studied in this work, and **L1PtCl** reported by Sil et al.^30^ Structure **L1PtCl** has been redrawn with permission from ref 30. Copyright 2025, John Wiley and Sons.

The *meta* complexes (**Pt** ^**m**^ and **Pd** ^**m**^) were further characterized by single crystal X-ray diffraction (SCXRD). Two symmetrically independent molecules of **Pt** ^**m**^ (A and B) and molecule **Pd** ^**m**^ are isostructural and have similar conformations (Figures 2a and S13). The metal atom adopts a distorted square coordination (planar within experimental error). Bond distances in **Pd** ^**m**^ (see Table S2) are similar to those in unsubstituted (3,5-bis­(pyridin-2-yl)­phenyl)­PdCl and its 5-substituted derivatives (which are also planar),^21^ the M–N bonds in **Pt** ^**m**^ are ca. 0.03 Å shorter than in **Pd** ^**m**^. In contrast, the platinum and palladium N^C^N complexes with bulky fused systems at the pyridine rings, studied by Soro et al.,^34^ strongly deviate from planarity, the Pd–Cl and Pt–Cl bonds tilting out of the chelate plane by 27.5° and 24.4°, respectively. In the latter complexes, M–N bonds are 0.07 Å longer than the corresponding bonds in **Pt** ^**m**^ and **Pd** ^**m**^. The M–C and M–Cl distances are similar in all the above-mentioned complexes.

**Figure 2:**
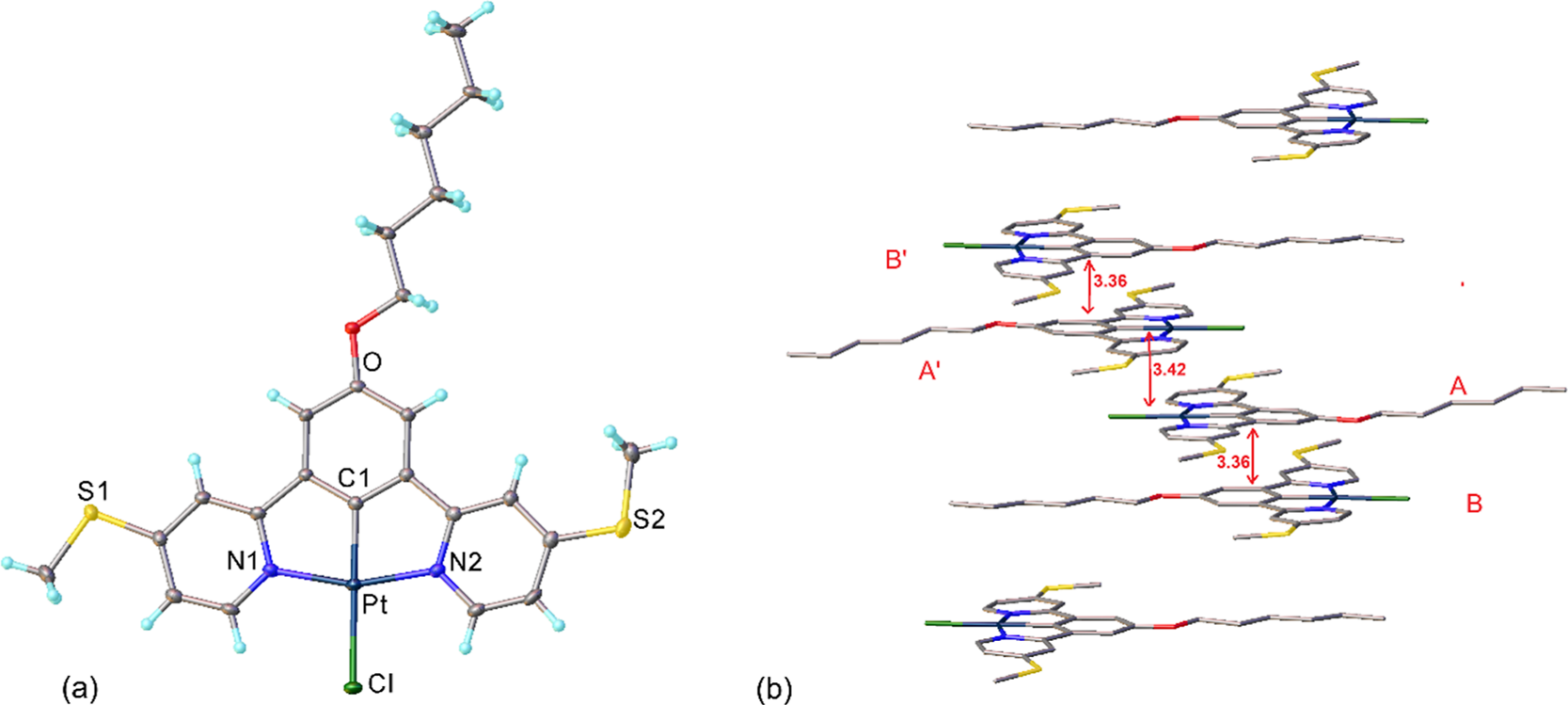
X-ray molecular structure (a) and π–π stacking (b) of **Pt** ^**m**^. Atomic displacement ellipsoids are shown at 50% probability level (a), hydrogen atoms are omitted for clarity.

The entire **Pd** ^**m**^ and **Pd** ^**m**^ molecules are approximately planar: the non-H atoms deviate from the mean plane by 0.13 Å (**Pt** ^**m**^, A), 0.08 Å (**Pt** ^**m**^, B), or 0.16 Å (**Pd** ^**m**^), and in the five-ring core by only 0.04 Å (**Pt** ^**m**^) or 0.06 Å **(Pd** ^**m**^). Despite their structural similarity, the crystal packing of **Pt** ^**m**^ and **Pd** ^**m**^ differs substantially (Figures 2b, S14 and S15). **Pd** ^**m**^ molecules form a centrosymmetric dimer, in which the molecular cores are π–π stacked with interplanar separation of 3.38 Å. Adjacent dimers are separated by a layer of aliphatic groups involving two *n*-hexyl chains and one (thio)­methyl group. A similar motif was observed for Pd­(N^C^N)Cl norvaline derivative.^35^ The structure of **Pt** ^**m**^ contains centrosymmetric tetramers (B···A···A′···B′), also separated by single layers of *n*-hexyl groups. The cores of molecule A and its inversion equivalent (rigorously parallel) are separated by 3.42 Å, those of A and B (parallel within 1.7°) by 3.36 Å, typical for π–π interactions.^36^

### Molecular Conductance Measurements

The molecular junctions were formed using a home-built scanning tunnelling microscope (STM) at ambient conditions.^37^ The STM tip was a mechanically cut 0.25 mm diameter gold wire (Goodfellow). The sample consisted of a preannealed gold surface (Arrandee) on which the compounds were deposited either from a ∼1 mM solution in dichloromethane and air-dried, or as a drop of ∼1 mM solution in mesitylene (Figure S21 in Supporting Information and Figure 3g,h). Concentration-dependence (0.5, 1.0, and 2.0 mM in dichloromethane) was studied for **Pt** ^**p**^ (Figure S22). After contact, the tip was pulled up so that the contact breaks enabling a single molecule to connect to the tip and the gold surface which act as electrodes: this is the so-called STM-break junction technique.^38,39^ The current was recorded and plotted against distance to determine a single-molecule junction at the conductance plateau (inset Figures S18–S20 in Supporting Information). After 7000 measurements 1D conductance (Figure 3) and 2D conductance vs distance histograms (Figures S18–S20 in Supporting Information) were generated from the 100 to 300 selected traces showing molecular plateaus, excluding traces showing clean retractions (i.e., no molecular junction formation), saturation of the electrical signal, or nonwell-defined breaks. The *k*-means clustering algorithm^40,41^ was used to separate traces with similar conductance, resulting in a single conductance peak in the histogram. The data are introduced as linear vectors, created by appending the rows of a matrix constructed from the 2D-histogram with 40 bins between −1 and −6 log­(*G*/*G*_0_) values and 30 bins between 0 and 2.5 nm of displacement. Another vector is also appended from a 1D histogram with 100 bins spanning −1 to −6.5 log­(*G*/*G*_0_) values. Then, a variable number of clusters, up to 6, are separated, some elucidating different conductance behaviors and some formed by tunnel traces. The clusters with a molecular feature are reanalyzed to remove remaining tunnel traces. All clusters containing traces with molecular features are combined, and the algorithm is applied again, since clusters describing similar conductance behavior may have been separated, and some traces may have been misclassified. The separation is now performed based on the number of conductance classes expected from observing similar clusters along the process. From these two classes, with different conductance values and recorded break-off (BO) distances for the ligands or complexes, high- and low-conductance classes (HC and LC) were assigned.

**Figure 3:**
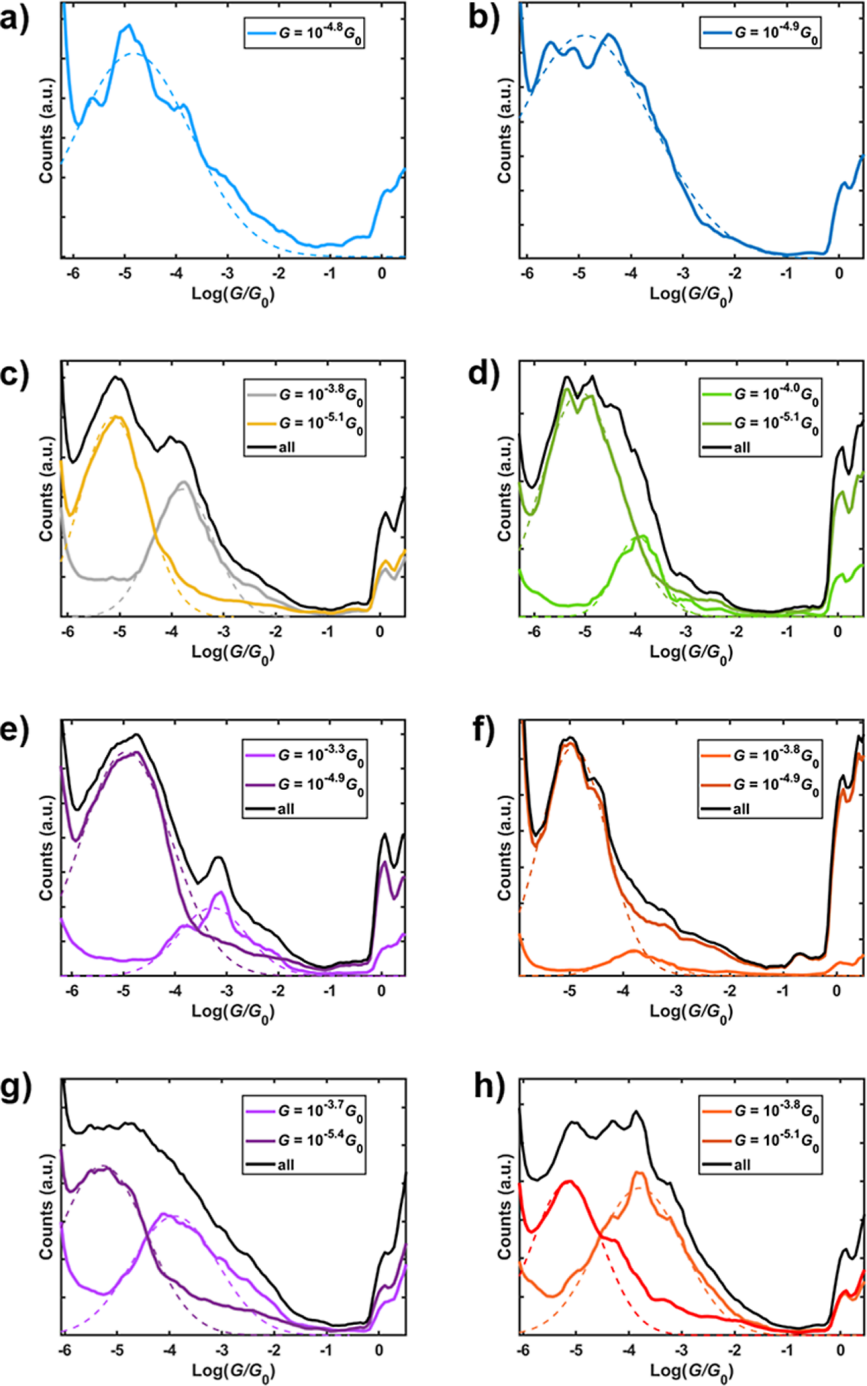
1D conductance histograms for compounds (a) **L** ^**m**^H, (b) **L** ^**p**^H, (c) **Pd** ^**m**^, (d) **Pd** ^**p**^, (e) **Pt** ^**m**^, and (f) **Pt** ^**p**^ measured in air, and (g) **Pt** ^**m**^ and (h) **Pt** ^**p**^ measured in solvent. Gaussian-fitted dashed lines highlight the HC and LC peaks with the conductance determined from the mean given in the respective legend.

Initially, the break-off (BO) distances were examined to understand the nature of the junction associated with each conductance feature. For this, the length of each plateau was considered to be the higher distance point within a conductance range of *G*_m_ ± 2σ, where *G*_m_ is the mean conductance of the Gaussian fitting of the conductance histogram (Figure 3) and σ is the standard deviation. This length histogram was fitted to a Lorentzian distribution from which the mean length was determined as the location parameter with an uncertainty described by the scale parameter (Figure S23 in Supporting Information). Table 1 provides the BO distances of all data sets which fit with the 2D histograms in the Supporting Information (Figures S18–S20). Regarding the junctions formed via thiomethyl contacts, according to a combination of density functional theory (DFT) calculations (see below) and X-ray crystallographic data (see Supporting Information, Table S6), **L** ^**p**^H has an intramolecular S···S distance of 1.22 nm which should be similar for both **Pd** ^**p**^ and **Pt** ^**p**^. However, the rotation of the pyridyl groups in the free ligand **L** ^**m**^H results in the S···S distance of 0.73–1.26 nm, but the metal coordination stops the ring rotation, enforces planarity on the triaryl ligand system, thereby fixing the S···S distance at 1.26 nm.

**Table 1:** Break Off Distances (nm) for All Data Sets^a^

molecule	**L** ^**m**^H	**L** ^**p**^H	**Pd** ^**m**^	**Pd** ^**p**^	**Pt** ^**m**^	**Pt** ^**p**^
HC	1.16 ± 0.35	1.20 ± 0.41	0.89 ± 0.21	0.51 ± 0.21	1.02 ± 0.17	0.63 ± 0.15
LC	-	-	0.96 ± 0.24	0.75 ± 0.23	1.04 ± 0.29	0.83 ± 0.25

a The uncertainty is the scale parameter of the distribution. The rows correspond to the high and low conductance classes (HC and LC) of each molecule as columns. The 2D conductance vs distance histograms for comparison can be found in Supporting Information Figures S18–S20.

A strongly dominant class (in air and solvent) can be fitted with conductance values of **L** ^**m**^H = −4.8 log­(*G*/*G*_0_) and **L** ^**p**^H = −4.9 log­(*G*/*G*_0_) in the conductance histograms of the free ligands. These values are comparable to the recent work by Sil et al. on the (*para*-connected) free ligand of complex **L** ^**1**^ **PtCl** (Figure 1) [namely 6,6′-(5-(*tert*-butyl)-1,3-phenylene)­bis­(3-(methylthio)­pyridine) (**L** ^**1**^H)],^30^ a structurally similar molecule to **L** ^**p**^H, for which the S···S contacted junction had a low conductance value of ∼−5.0 log­(*G*/*G*_0_). Despite the similarity of the systems, Sil et al. also observed a weak higher conductance feature at ∼−2.4 log­(*G*/*G*_0_) attributed to contact via the pyridine (i.e., an N···S junction):^30^ this feature was not observed in our study for either of the free ligands **L** ^**m**^H or **L** ^**p**^H. To assess the possibility of such interactions a monolayer of **L** ^**m**^H was prepared by immersing a gold substrate in a 1 mM solution in dichloromethane for 48 h and evaluated by X-ray photoelectron spectroscopy (XPS). Figure S24 shows the XPS spectra for the powder and a SAM of **L** ^**m**^H in the N 1s region. The powder showed only a peak at 398.2 eV attributed to the free pyridyl groups, whereas the SAM of **L** ^**m**^H showed two peaks in the N 1s region: one at 398.2 eV similar to the powdered sample and another peak at 399.1 eV attributed to the interaction of some of the pyridyl groups with the gold substrate.^29,42^ The absence of N···S junctions in the present study suggests that although the free ligands are capable of contacting the gold electrodes via the pyridyl groups,^43^ this is not favorable, presumably because the central phenylene ring sterically hinders the nitrogen lone pair.

All four metal complexes **Pd** ^**p**^, **Pd** ^**m**^, **Pt** ^**p**^, and **Pt** ^**m**^ displayed two conductance classes. The BO distances were shorter than those of the free ligands, attributed to the increased rigidity of the complexes causing the junction to break before fully extending. However, both **Pd** ^**m**^ and **Pt** ^**m**^ have longer BO distances than **Pd** ^**p**^ and **Pt** ^**p**^ consistent with the difference in the S···S distance (see Supporting Information, Table S6). There were significant differences in the ratio between the HC and LC classes (Table S5 in Supporting Information), e.g., the HC class accounts for 10.1% of the traces for **Pt** ^**p**^ in air (Figure 3f) while in mesitylene (Figure 3h), it accounts for 43.5%. The suppression of the LC feature in the solvent suggests that this junction configuration is associated with an intermolecular interaction. Comparing the conductance values to existing literature, the platinum­(II) complex **L** ^**1**^ **PtCl** (Figure 1) (a structurally similar analog of **Pt** ^**p**^) has an S···S conductance value of −3.9 log­(*G*/*G*_0_)^30^ very similar to the measured HC value of **Pt** ^**p**^ (−3.8 log­(*G*/*G*_0_)) indicating that the HC classes of the metal complexes are associated with S···S contacted junctions, as proposed previously for **L** ^**1**^ **PtCl**.^30^ Therefore, the LC features are likely to be π–π stacked dimers which is supported by the trends observed from the calculations and is in agreement with the experimental observation of π–π stacking in X-ray crystallographic data (Figures 2b, S14 and S15) and the longer BO distances in Table 1. Interestingly, Sil et al. highlighted the significant role of metal-contacted junctions with **L** ^**1**^ **PtCl** (i.e., < with a Pt–Au intermetallic bond) based on the limited extension of the HC plateau at −2.1 log­(*G*/*G*_0_) (0.70 ± 0.06 nm).^30^ However, in contrast, for our four metal complexes we observed few traces in the conductance region attributed to this contact geometry under the same conditions as Sil et al.^30^

There is precedent for in situ bonding of electrode gold atoms into coordination chains of organic ligands (e.g., triazole units) within a molecular junction.^44^ To shed more light on the possibility of Pt–Au or Pd–Au contacted junctions in our systems, a monolayer of each complex **Pt** ^**m**^, **Pd** ^**m**^, **Pt** ^**p**^ or **Pd** ^**p**^ was deposited on a gold surface for XPS analysis. Self-assembled monolayers (SAMs) were prepared by immersing the gold substrate in a 1 mM solution in DCM for 48 h. There was a doublet at 72.0 and 75.3 eV assigned to (4f_7/2_) and (4f_5/2_), respectively, in the powdered sample of **Pt** ^**m**^ in the Pt 4f region (Figure 4). On the contrary, the XPS data from SAMs of these platinum complexes are more convoluted. The spectra showed a comparable doublet peak as observed for the powder (at 72.1 and 75.2 eV, respectively) and a new doublet at higher binding energies of 73.0 and 76.0 eV attributed to a Pt–Au interaction, suggesting that these platinum complexes can form junctions via the metal center. Meanwhile for the palladium complexes, the XPS data from SAMs in the Au 4d/Pd 3d region showed a peak at 342.4 eV attributed to Pd­(2+), 3d_3/2_, and a peak at 340.2 eV attributed to Pd(0), 3d_3/2_, (Figure 4).^29,45^ The respective Pd 3d_5/2_ peaks for the SAMs were obscured by the Au 4d_5/2_ peak due to the gold substrate. These results indicate that there is a partial interchange of the palladium by gold, therefore the pyridyl groups can directly contact the gold substrate, potentially accounting for the high conductance shoulder of the HC class at ∼−2.5 to −3.0 log­(*G*/*G*_0_) observed in some of the complexes (Figures 3 and S21).

**Figure 4:**
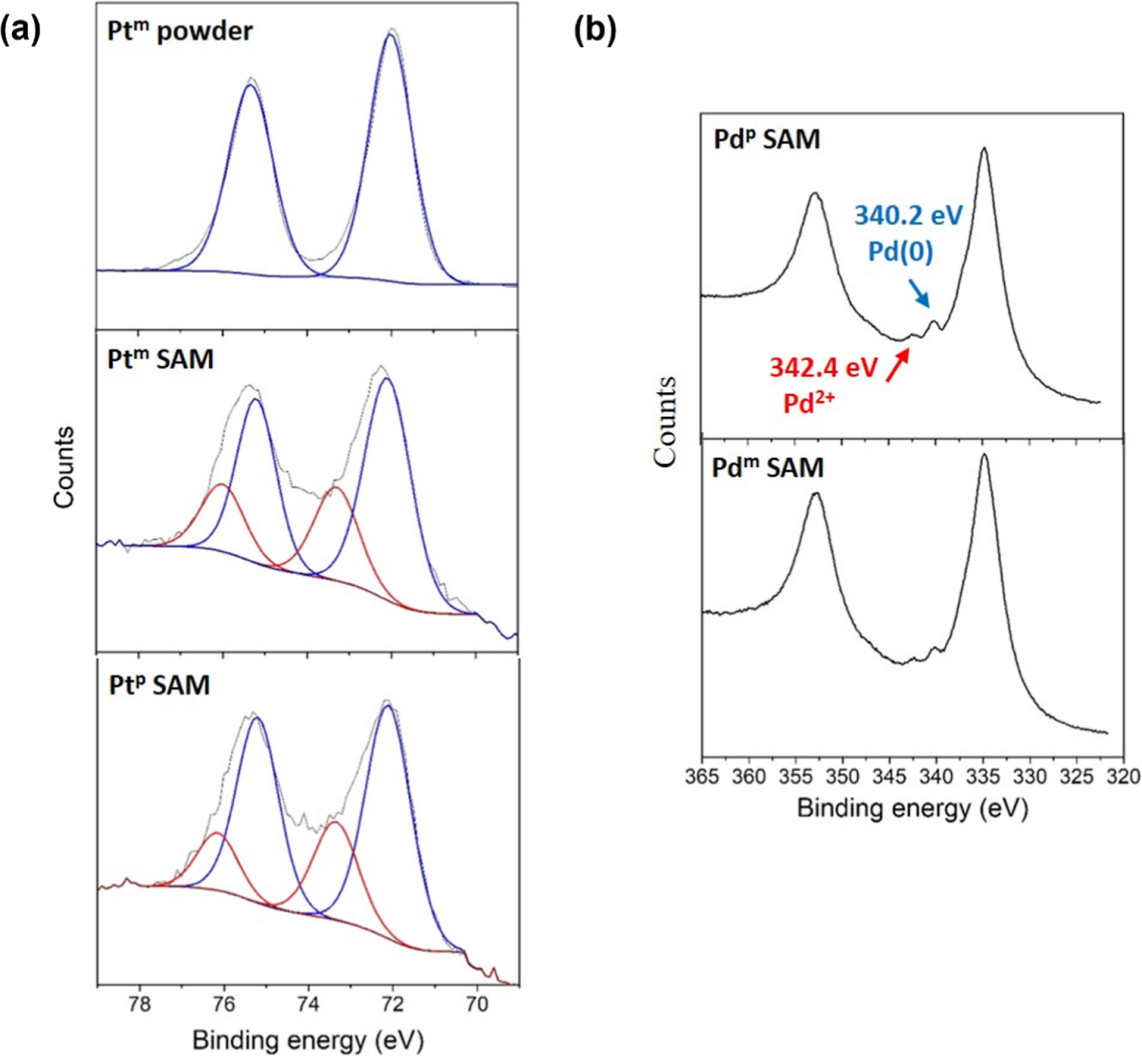
(a) XPS spectra in the Pt 4f region corresponding to **Pt** ^**m**^ powder and SAM and **Pt** ^**p**^ SAM. (b) XPS spectra in the Au 4d/Pd 3d region corresponding to **Pd** ^**p**^ and **Pd** ^**m**^ SAMs.

However, despite the behavior observed by XPS, few conductive traces can be attributed to a metal contact junction for any of the metal complexes in this study. To explain this discrepancy, it must be considered that the SAMs used for the XPS measurements are a static environment where the molecules are arranged into the most energetically favorable assembly, whereas a molecular junction is a dynamic environment where both the energy and kinetics play a role in the junction configuration. Therefore, we propose that there must be an interaction within the molecules that competes with the M–Au interaction, most likely π–π stacking based on the planar nature of the molecules. The formation of π-stacked dimers, accounting for the observed LC class, would hinder M–Au interactions. It is interesting that Sil et al. specifically discounted π-stacked dimers in their work due the *t*-Bu substituent on complex **L** ^**1**^ **PtCl** hindering this arrangement, and the lack of features at the larger electrode separation generally found in dimers.^30^

Having established the junction geometry of each conductance class, the impact of the molecule’s structure was then examined. From this point onward, unless otherwise stated, the discussion refers to the S···S contacted junctions. First, the measured conductance of both of the free ligands is very similar (**L** ^**m**^H 10^–4.8^ *G*_0_; **L** ^**p**^H 10^–4.9^ *G*_0_; Figure 3a,b) as expected, due to the dominant effect of the *meta* connection at the central phenylene ring,^46^ and there is a significant increase in conductance upon metal coordination, with the platinum complexes having a higher conductance than their palladium analogs, e.g., **L** ^**m**^H = −4.8 log­(*G*/*G*_0_), **Pd** ^**m**^ = −3.8 log­(*G*/*G*_0_), and **Pt** ^**m**^ = −3.3 log­(*G*/*G*_0_). This trend correlates with the experimental Δ*E*(HOMO–LUMO) which is significantly larger for the free ligands compared with their metal-complexed analogs, by 0.68–0.96 eV: e.g., **L** ^**m**^H = 3.58 eV, **Pd** ^**m**^ = 2.91 eV, and **Pt** ^**m**^ = 2.62 eV, based on electronic absorbance spectra in solution (Table S3 and Figure S16; see Supporting Information for full details), consistent with the behavior reported by Ponce et al. and Chelli et al.,^31,32^ i.e., a smaller Δ*E*(HOMO–LUMO) translates to higher conductance. It is seen in experiments and theory that Δ*E*(HOMO–LUMO) is smaller with Pt coordination compared to Pd for both *meta* and *para* isomers. A similar trend was observed for the low conductance π-stacked dimers of the metal complexes (Figure 3c,e); the conductance for the ligand dimers (if present) was too low for detection.

Second, comparing the linkage isomerism (i.e., *para* vs *meta*-connected thiomethyl groups), it is notable that each *meta* complex has a higher conductance than its *para*-connected analog, in contrast to the typically observed behavior for purely organic systems where destructive quantum interference effects operate through *meta* π-conjugated rings.^46–50^ For consistency, the *meta* and *para* nomenclature of the free ligands is retained in their respective complexes. Therefore, in the complexes the [anchor···N–M–N···anchor] transmission pathway is *para* at both terminal pyridyl rings, relative to the metal center. For the ligands (**L** ^**p**^H and **L** ^**m**^H) and the palladium complexes (**Pd** ^**p**^ and **Pd** ^**m**^) the conductance differs by only 0.1 log­(*G*/*G*_0_) (Figure 3a,b) and 0.2 log­(*G*/*G*_0_) (Figure 3c,d) respectively, but this becomes more significant for the platinum complexes (**Pt** ^**p**^ and **Pt** ^**m**^) where the difference is 0.5 log­(*G*/*G*_0_) (Figure 3e,f). We considered the possible impact of molecular length on the *meta* vs *para* conductance behavior. **Pd** ^**p**^ and **Pt** ^**p**^ have near identical S···S distances, as do **Pd** ^**m**^ and **Pt** ^**m**^ (Table S6), but **Pd** ^**p**^ and **Pd** ^**m**^ have a 0.2 log­(*G*/*G*_0_) difference in conductance while **Pt** ^**p**^ and **Pt** ^**m**^ have a 0.5 log­(*G*/*G*_0_) difference (Figure 3). If this was purely due to a length dependence, then it would be expected that the conductance difference would be the same for both metals, therefore, molecular length is not believed to be a significant factor. An explanation is provided by the theoretical simulations below.

### Theoretical Simulations

Quantum transport calculations through the molecules were performed to understand the experimental results. First, the ground-state geometries of each molecule in the gas phase and in the junctions between two gold electrodes were determined using the SIESTA^51^ implementation of density functional theory (DFT). Then, the ground-state Hamiltonian and overlap matrices were obtained from DFT and combined with the transport code GOLLUM^52^ to calculate the transmission coefficient *T*(*E*) for each molecule between the gold electrodes (see Section S9 in Supporting Information for more details). The simulated transmission functions *T*(*E*) were analyzed to extract key conductance features and their dependence on the molecular configurations and junction geometries. Simulations were conducted for both free ligands (**L** ^**m**^H, **L** ^**p**^H) and their corresponding palladium (**Pd** ^**m**^, **Pd** ^**p**^) and platinum (**Pt** ^**m**^, **Pt** ^**p**^) complexes in molecular junctions (Figures S30–S36) to align with experimental conditions. The computed conductance features were benchmarked against experimental 1D conductance histograms. Although a quantitative fitting of the conductance data could not be achieved, qualitatively, the same trends were shown for the HOMO–LUMO gap data (see Supporting Information, Table S7) and relative conductance between ligands and their analogous metal complexes, i.e., the compounds with a lower HOMO–LUMO gap had a higher conductance. Based on the break-off distances (Table 1) and comparisons to previous systems,^30^ it was determined that the HC feature is associated with an S···S contacted junction, while the LC feature is associated with a π–π stacked geometry. The conductance is LUMO-dominated based on negative Seebeck coefficients measured for **Pd** ^**m**^ (−3.1 μV/K) and **Pd** ^**p**^ (−1.2 μV/K) (Figure S27) and occurs in a region removed from significant QI features. Previous studies with thiomethyl anchors demonstrate that a donor–acceptor dative bond is formed through delocalization of the sulfur lone pair to an undercoordinated gold atom,^53,54^ leading to either HOMO- or LUMO-dominated transport, depending on the molecular backbone.^55–58^ Although an exact fitting of the Fermi energy (*E*_F_) could not be achieved, we can narrow the discussion of the transmission curves that fit this criterion, approximately −1.0–0 *E*_F_ (eV).

Figure 5 shows the DFT conductance at room temperature for ligand (**L** ^**m**^H) and metal-complexes (**Pd** ^**m**^, **Pd** ^**p**^ and **Pt** ^**p**^); ligand **L** ^**p**^H and **Pt** ^**m**^ are shown in Figures S37 and S40, respectively. Our calculations reveal that the conductance increases when comparing ligands in a type I contact geometry (each sulfur atom making contact with a single gold atom of each electrode). **L** ^**p**^H has higher conductance than **L** ^**m**^H consistent with solution conductance values [see Supporting Information, Figure S21, **L** ^**m**^H *G* = −5.2 log­(*G*/*G*_0_) and **L** ^**p**^H *G* = −4.6 log­(*G*/*G*_0_)] and typical of *meta*/*para* organic molecules.^46^ However, the relative conductance between ligands is very dependent on *E*_F_ for a type II junction (each sulfur atom making contact with two gold atoms of each electrode) (Figure S38) and may account for the negligible difference in conductance measurements in air [**L** ^**p**^H *G* = −4.9 log­(*G*/*G*_0_) and **L** ^**m**^H *G* = −4.8 log­(*G*/*G*_0_)] (Figure 3a). For the metal complexes the calculated conductance increases relative to their free ligand precursors, consistent with the conductance data collected in solution and in air. Previously, Ponce et al., Chelli et al., and Bastante et al. observed that metal coordination increases conductance due to the contraction of the HOMO–LUMO energy gap.^31,32,59^ However, in the aforementioned examples, the *para* complexes maintained a higher conductance than their *meta* analogs, in contrast to that observed for the metal complexes discussed in this paper; here the *meta* complexes have a calculated conductance higher than their *para* analogs for all *E*_F_ values_,_ consistent with the measured conductance. This can be explained by the significant amount of orbital overlap occurring between metal d-orbitals and phenylate π-orbitals (Figures S44 and S45), similar to that reported by Sotoyama et al. for Pt­(N^C^N)Cl coordination complexes.^60^ Such orbital overlap in the present series of complexes does not occur in the metal complexes of the imine-based ligand systems previously discussed.^32,60^ Despite the calculated and measured differences in the HOMO–LUMO energy gap resulting from changing the metal from palladium to platinum, there is a negligible difference in the calculated electrical conductance for all *E*_F_ values, consistent with the measured conductance.

**Figure 5:**
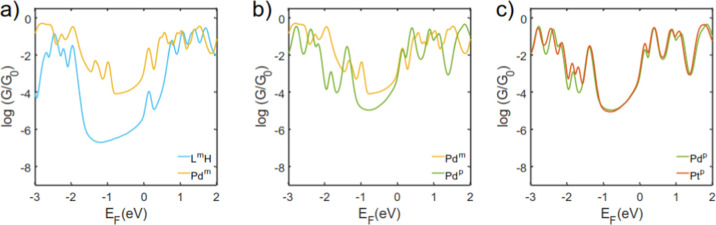
DFT conductance calculations at room temperature. (a) **L** ^**m**^H, **Pd** ^**m**^ (b) **Pd** ^**m**^, **Pd** ^**p**^ and (c) **Pd** ^**p**^, **Pt** ^**p**^.

While an antiresonance is generally expected for *meta*-connected molecules (often considered a key indication of destructive quantum interference, DQI), especially in ligands where transmission is dominated by a well-defined *meta*-connected benzene core,^46^ the transmissions in Figures S37–S39 do not show a clear antiresonance feature. The antiresonance feature is typically expected in molecules where transport is primarily governed by π-orbitals and the active part of the molecule contributing to QI (e.g., the molecular core) is weakly coupled to the electrodes. In the molecules studied here, the *meta*/*para* connection that is varied is on the anchors, meaning they are not electronically isolated from the electrodes. Additionally, other orbitals, such as σ-orbitals, can contribute to transport due to the small molecular size. Therefore, the antiresonance feature is expected to be washed-out which is consistent with other studies.^61^ Nonetheless, the manifestation of the DQI effect is apparent in the lower amplitude of transmission for the *meta*-connected ligand (**L** ^**m**^H) compared to the *para*-connected isomer (**L** ^**p**^H).

An examination of the π–π stacked dimers shows their lower conductance relative to their monomer analogs due to weaker intermolecular electronic coupling compared to thiomethyl-electrode coupling (Figure S42). However, similar trends to the monomers were observed with the *meta* and *para* complexes having similar electronic conductance and the impact of the metal being negligible, consistent with the measured values.

Finally, although such a feature did not play a role in the measurement of these molecules, the *meta* complexes (**Pd** ^**m**^ and **Pt** ^**m**^) display a Fano feature at −1.0 eV *E*–*E*_F_ (Figure S40), which does not appear to be present for the *para* analogs **Pd** ^**p**^ and **Pt** ^**p**^. This provides a future avenue for exploration, given that it is associated with the HOMO orbitals of the system that consist of d­(M)–π orbital mixing.

## Conclusions

Two new 3,5-bis­(pyridin-2-yl)­phenyl (N^C^N)-based ligands and their palladium and platinum chloride complexes were synthesized with terminal thiomethyl contact groups in the *para* or *meta* positions of the pyridyl groups. A combination of XPS studies and comparative conductance data in air/solvent showed that although the metal complexes are capable of direct interactions between the metal center and the gold electrode, competition from face-to-face π–π stacking prevented such contacts from occurring in the molecular junction. The conductance of the *meta*-contacted ligand **L** ^**m**^H was essentially the same as its *para*-analog **L** ^**p**^H, due to the prominent effect of *meta*-substitution at the central phenylene ring. Metal coordination significantly enhanced the conductance relative to the corresponding free ligands, and, most notably, the complexes that incorporate ligands substituted with thiomethyl groups in *meta* positions relative to the pyridine-benzene linkages **Pt** ^**m**^ and **Pd** ^**m**^ had a significantly higher conductance than their *para*-analogs **Pt** ^**p**^ and **Pd** ^**p**^. This is attributed to the extent of orbital mixing between the metal center and the ligand π-orbitals resulting in a dominant [anchor···N–M–N···anchor] transmission pathway that is *para* at both terminal pyridyl rings, relative to the metal center. Overall, our study gives new insights into the fascinating effects that metal coordination to organic ligands (and the associated d­(M)–π orbital mixing) can exert on charge transport pathways in single-molecule junctions. The results should stimulate further studies of molecular junctions that incorporate complexes with a range of metal and ligand combinations.

## Supplementary Material



## Data Availability

The data associated with this article is available in the manuscript and Supporting Information files.

## Notes

Experimental procedures, NMR spectra, photophysical data, conductance and Seebeck measurements, XPS data, computational methods and data, crystallographic data for compounds **Pd** ^**m**^ and **Pt** ^**m**^ (CCDC 2334297 and 2334298) (PDF)
