# Supplementary material for: Experimental and Theoretical Studies of Isomeric Metal (N^C^N)Cl Coordination Complexes (Metal = Pt, Pd) with Multiple Conductance Pathways in Single-Molecule Junctions
Source: J Phys Chem C Nanomater Interfaces. 2026 Feb 6;130(7):2763–72. doi: 10.1021/acs.jpcc.5c07119 (PMC12927009; doi:10.1021/acs.jpcc.5c07119)
Supplement: Supplementary file 1 [file jp5c07119_si_001.pdf]

SUPPORTING INFORMATION

Experimental and Theoretical Studies of Isomeric Metal(N^C^N)Cl Coordination Complexes (Metal = Pt, Pd) with Multiple Conduction Pathways in Single-Molecule Junctions

Authors:

Pablo Bastante[1], Ross J. Davidson[2], Yahia Chelli[3], Abdalghani H. S. Daaoub[3], Pilar Cea[4,5], Santiago Martin[4,5], Andrei S. Batsanov[2], Sara Sangtarash[3], Hatef Sadeghi[3]*, Martin R. Bryce[2]*, Nicolas Agrait[1]*

Affiliations:

[1]*Departamento de Física de la Materia Condensada C-III, and Instituto Universitario de Ciencia de Materiales "Nicolás Cabrera", Universidad Autónoma de Madrid, E-28049 Madrid, Spain.*

[2] *Department of Chemistry, Durham University, Durham, DH1 3LE, U.K.*

[3]*Quantum Device Modelling Group, School of Engineering, University of Warwick, Coventry CV4 7AL, U.K.*

[4]*Instituto de Nanociencia y Materiales de Aragón (INMA), CSIC-Universidad de Zaragoza, 50009, Zaragoza, Spain.*

[5]*Departamento de Química Física, Universidad de Zaragoza, 50009, Zaragoza, Spain and Laboratorio de Microscopias Avanzadas (LMA), Universidad de Zaragoza, 50018, Zaragoza, Spain.*

Table of Contents

S1. Synthesis of reported compounds S3

S2. NMR spectra of reported compounds S9

S3. X-ray Crystallography S15

S4. Photophysical data for reported compounds S21

S5. STM-BJ measurements S25

S6. Junction length determination S30

S7. XPS Results S31

S8. Thermopower measurements S32

S9. Theory S33

References S45

S1. Synthesis of reported compounds

Instrumentation. NMR spectra were recorded in deuterated solvent solutions using a Varian VNMRS-700 spectrometer and referenced against solvent resonances (^{1}H, ^{13}C). ASAP data were recorded using a Xevo QTOF (Waters) high resolution, accurate mass tandem mass spectrometer equipped with atmospheric pressure gas chromatography (APGC) and an atmospheric solids analysis probe (ASAP). Microanalyses were performed by the Elemental Microanalysis Service, Durham University (UK).

General details. The compounds 2,2'-(5-(hexyloxy)-1,3-phenylene)bis(4,4,5,5-tetramethyl-1,3,2-dioxaborolane), 2-bromo-5-(methylthio)pyridine,[1] and 2-bromo-4-(methylthio)pyridine[2] were prepared according to published methods. All other chemicals were sourced from standard chemical suppliers.

General ligand synthesis

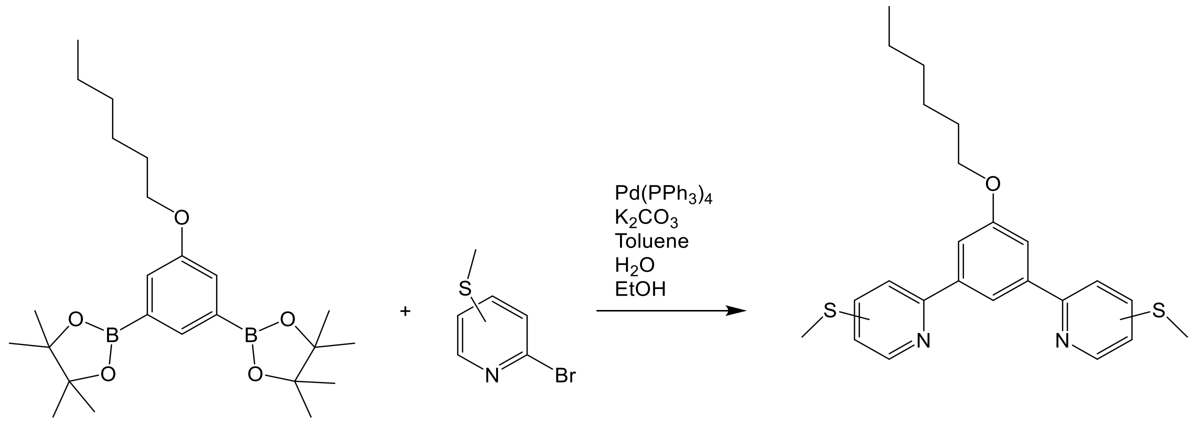

$Pd(PPh_3)_4$ (5 mmol%) was added to a solution containing 2-bromo-5-(methylthio)pyridine or 2-bromo-4-(methylthio)pyridine (1.22 g, 6.06 mmol), 2,2'-(5-(hexyloxy)-1,3-phenylene)bis(4,4,5,5-tetramethyl-1,3,2-dioxaborolane) (1.30 g, 3.03 mmol), K_2CO_3 (1.67 g,

12.12 mmol), toluene (70 mL), H_2O (8 mL) and EtOH (10 mL). The solution was then degassed by three freeze–pump–thaw cycles. The reaction mixture was heated to reflux for 48 hours before the solvent was removed *in vacuo*, then the residue was dissolved in DCM and filtered. The filtrate was collected, the solvent was removed and the residue was purified by silica chromatography eluted by a solvent gradient from neat DCM to DCM:EtOAc (1:1 v/v). A white oil that solidified upon standing was obtained.

2,2'-(5-(hexyloxy)-1,3-phenylene)bis(4-(methylthio)pyridine) ($\mathbf{L^m}$H) [from 2-bromo-4-(methylthio)pyridine] **Yield**: 1.04 g (81%). **^{1}H NMR** (700 MHz; CD_2Cl_2): δ_H 8.48 (d, $^3J_{HH}$ = 5.3 Hz, 2H, H_e), 8.16 (s, 1H, H_a), 7.64 (d, $^4J_{HH}$ = 1.9 Hz, 2H, H_c), 7.61 (d, $^4J_{HH}$ = 1.3 Hz, 2H, H_b), 7.09 (dd, $^3J_{HH}$ = 5.3 Hz, $^4J_{HH}$ = 1.9 Hz, 2H, H_d), 4.13 (t, $^3J_{HH}$ = 6.5 Hz, 2H, H_g), 2.57 (s, 6H, H_f), 1.84 (p, $^3J_{HH}$ = 6.8 Hz, 2H, H_h), 1.52 (p, $^3J_{HH}$ = 7.3 Hz, 2H, H_i), 1.41-1.35 (m, 4H, H_j+H_k), 0.93 (t, $^3J_{HH}$ = 6.7 Hz, 3H, H_l) ppm. **^{13}C{^{1}H} NMR** (125 MHz; $CDCl_3$): δ_C 160.0, 156.3, 150.9, 148.9, 140.8, 118.3, 117.7, 116.8, 113.7, 68.3, 31.5, 29.2, 25.6, 22.6, 13.8, 13.7 ppm. **Acc-MS**(ASAP$^+$): *m/z* 425.1700 $[M+H]^+$ calcd. for $C_{24}H_{29}N_2S_2O$ *m/z* ($|\Delta m/z|$ = 1.0 ppm). **Anal. Calc.** for $C_{24}H_{28}N_2OS_2$: C, 67.89; H, 6.65; N, 6.60 %. **Found**: C, 67.37; H, 6.59; N, 6.52 %.

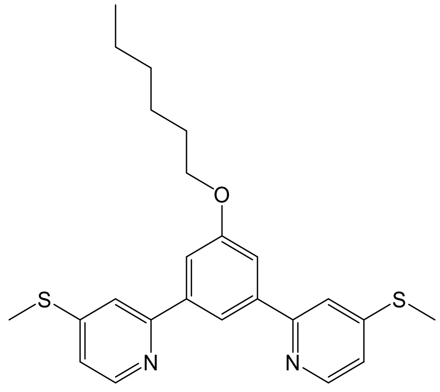

6,6′-(5-(hexyloxy)-1,3-phenylene)bis(3-(methylthio)pyridine)) (**L**^{p}H) [from 2-bromo-5-(methylthio)pyridine] **Yield**: 0.92 g (72%). **^{1}H NMR** (700 MHz; CD_2Cl_2): δ_H 8.58 (d, $^4J_{HH}$ = 2.5 Hz, 2H, H_e), 8.20 (t, $^4J_{HH}$ = 1.5 Hz, 1H, H_a), 7.78 (d, $^3J_{HH}$ = 8.4 Hz, 2H, H_c), 7.67 (dd, $^3J_{HH}$ = 8.4 Hz, $^4J_{HH}$ = 2.5 Hz, 2H, H_d), 7.60 (d, $^3J_{HH}$ = 7.60 Hz, 2H, H_b), 4.12 (t, $^3J_{HH}$ = 6.6 Hz, 2H, H_g), 2.56 (s, 6H, H_f), 1.85-1.82 (m, 2H, H_h), 1.54-1.49 (m, 2H, H_i), 1.40-1.36 (m, 4H, H_j+H_k), 0.94-0.91 (m, 3H, H_l) ppm. **^{13}C{^{1}H} NMR** (125 MHz; $CDCl_3$): δ_C 160.1, 153.3, 147.4, 140.4, 134.7, 134.3, 120.2, 116.9, 112.9, 68.3, 31.5, 29.2, 25.6, 22.6, 15.6, 13.7 ppm. **Acc-MS**(ASAP$^+$): *m/z* 425.1717 $[M+H]^+$ calcd. for $C_{24}H_{29}N_2S_2O$ *m/z* 425.1721 ($|\Delta m/z|$ = 0.9 ppm). **Anal. Calc.** for $C_{24}H_{28}N_2OS_2$: C, 67.89; H, 6.65; N, 6.60 %. **Found**: C, 67.61; H, 6.58; N, 6.46 %.

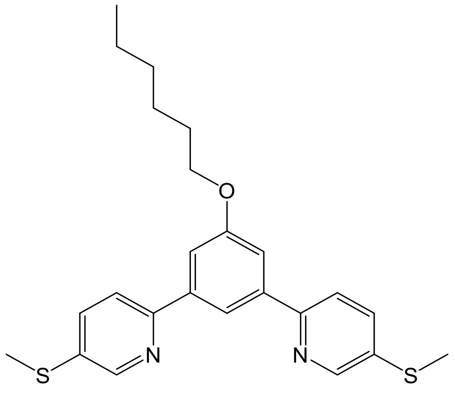

Metal coordination: general synthesis

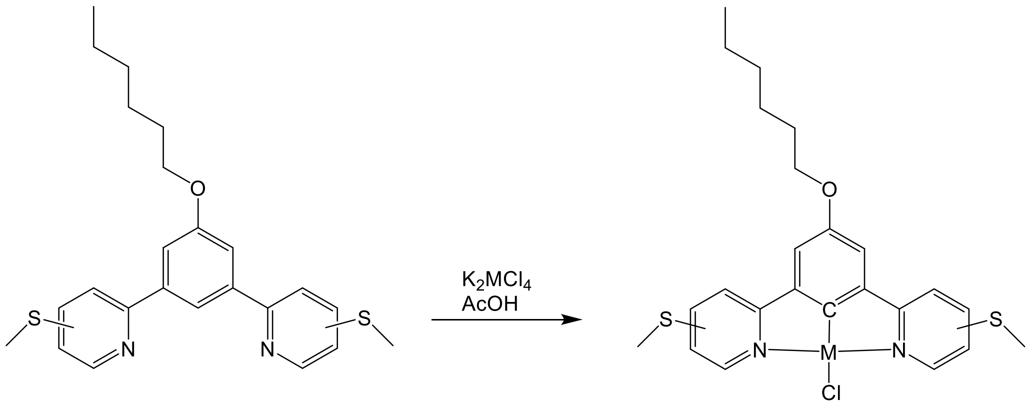

K_2MCl_4 (M = Pd or Pt, 0.24 mmol) was added to a solution containing **L**H (102 mg, 0.25 mmol) in AcOH (30 mL) and then heated to reflux under argon for 48 hours. The solution was cooled to room temperature forming a precipitate that was collected by filtration and washed thoroughly with methanol. Depending on the solubility, the complex was either extracted using hot $CHCl_3$ with the filtrate being collected (**Pd^m** and **Pt^m**) or washed with cold $CHCl_3$ with the precipitated collected (**Pd^p** and **Pt^p**).

PdL^{m}Cl (**Pd^m**). **Yield**: 85 mg (63%). **^{1}H NMR** (700 MHz; CD_2Cl_2): δ_H 8.60 (d, $^3J_{HH}$ = 6.0 Hz, 2H, H_a), 7.33 (d, $^4J_{HH}$ = 2.1 Hz, 2H, H_c), 6.94 (dd, $^3J_{HH}$ = 6.6 Hz, $^4J_{HH}$ = 2.1 Hz, 2H, H_b), 6.89 (s, 2H, H_d), 3.97 (t, $^3J_{HH}$ = 6.6 Hz, 2H, H_f), 2.59 (s, 6H, H_e), 1.80-1.75 (m, 2H, H_g), 1.51-1.46 (m, 2H, H_h), 1.39-1.36 (m, 4H, H_i+H_j), 0.95-0.93 (m, 3H, H_k) ppm. **^{13}C{^{1}H} NMR** (125 MHz; CD_2Cl_2): δ_C 163.4, 163.2, 156.9, 154.7, 150.5, 142.9, 118.2, 114.5, 110.7, 68.7, 31.6, 29.3, 25.7, 22.6, 13.9, 13.8 ppm. **Acc-MS**(ASAP$^+$): 529.0609 [M-Cl]$^+$ calcd. for $C_{24}H_{27}N_2OPdS_2$ *m/z* 529.0608 ($|\Delta m/z|$ = 0.1 ppm). **Anal. Calc.** for $C_{24}H_{27}ClN_2OPdS_2$: C, 50.98; H, 4.81; N, 4.95 %. **Found**: C, 50.72; H, 4.87; N, 4.52 %.

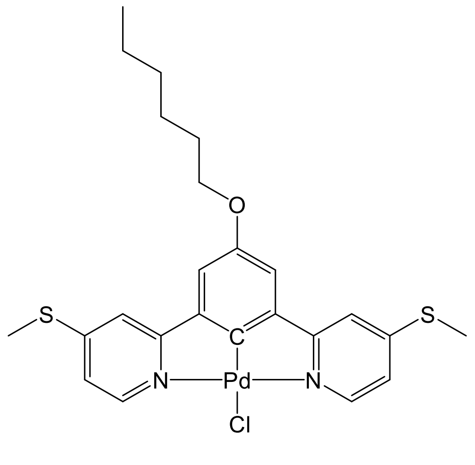

PtL^{m}Cl (**Pt^m**). **Yield**: 64 mg (41%). **^{1}H NMR** (700 MHz; CD_2Cl_2): δ_H 8.87 (d, $^3J_{HH}$ = 6.2 Hz, 2H, H_a), 7.36 (d, $^4J_{HH}$ = 2.2 Hz, 2H, H_c), 7.01-6.99 (m, 2H, H_b+H_d), 4.00 (t, $^3J_{HH}$ = 6.6 Hz, 2H, H_f), 2.60 (s, 6H, H_e), 1.82-1.77 (m, 2H, H_g), 1.41-1.36 (m, 4H, H_i+H_j), 0.95-0.93 (m, 3H, H_k) ppm. **^{13}C{^{1}H} NMR** (125 MHz; $CDCl_3$): δ_C 165.6, 156.3, 154.7, 152.6, 149.9, 140.7,

118.2, 114.9, 111.2, 68.9, 31.6, 29.4, 25.7, 22.6, 13.9, 13.8 ppm. **MS**(MALDI$^+$): *m/z* 1271.8 $[M_2\text{-Cl}]^+$, 1305.9 $[M_2]^+$. **Anal. Calc.** for $C_{24}H_{27}ClN_2OPtS_2\cdot½H_2O$: C, 43.47; H, 4.26; N, 4.22 %. **Found**: C, 43.56; H, 4.16; N, 4.03 %.

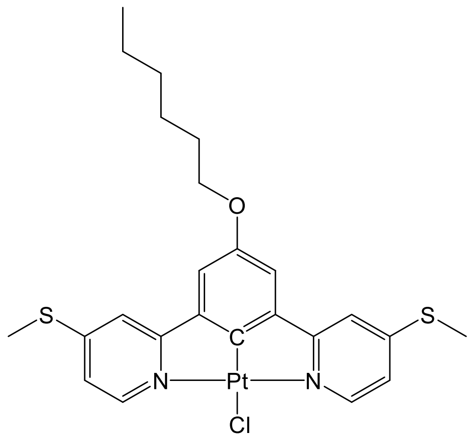

PdL^{p}Cl (**Pd^p**). **Yield**: 76 mg (56%). **^{1}H NMR** (700 MHz; $CDCl_3$): δ_H 8.84 (d, $^4J_{HH}$ = 2.4 Hz, 2H, H_a), 7.63 (dd, $^3J_{HH}$ = 8.4 Hz, $^4J_{HH}$ = 2.3 Hz, 2H, H_b), 7.43 (dd, $^4J_{HH}$ = 8.4 Hz, $^3J_{HH}$ = 0.7 Hz, 2H, H_c), 6.81 (s, 2H, H_d), 3.95 (t, $^4J_{HH}$ = 6.6 Hz, 2H, H_f), 2.56 (s, 6H, H_e), 1.76 (dq, $^3J_{HH}$ = 8.7 Hz, $^4J_{HH}$ = 6.7 Hz, 2H, H_g), 1.46 (p, $^4J_{HH}$ = 7.4 Hz, 2H, H_h), 1.36-1.33 (m, 4H, H_i+H_j), 0.92-0.90 (m, 3H, H_k) ppm. **^{13}C{^{1}H} NMR** (125 MHz; $CDCl_3$): δ_C 162.4, 160.8, 157.1, 149.3, 143.0, 136.0, 135.9, 118.4, 109.9, 68.7, 31.6, 29.3, 25.7, 22.6, 15.5, 14.0 ppm. **MS**(MALDI): *m/z* 1094.9 $[M_2\text{-Cl}]^+$. **Anal. Calc.** for $C_{24}H_{27}ClN_2OPdS_2$: C, 50.98; H, 4.81; N, 4.95 %. **Found**: C, 50.94; H, 4.82; N, 4.74 %.

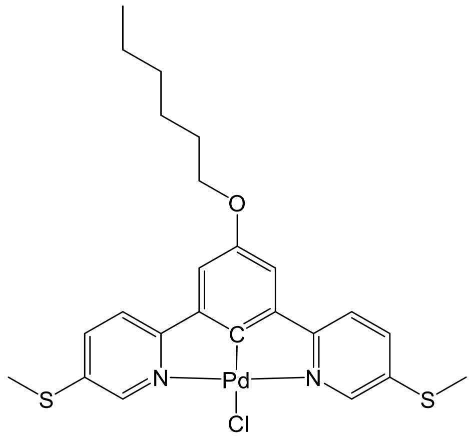

PtL^{P}Cl (**Pt^{P}**). **Yield**: 52 mg (35%). **^{1}H NMR** (700 MHz; $CDCl_3$): δ_H 9.13 (d, $^{4}J_{HH}$ = 2.2 Hz, 2H, H_a), 7.72 (dd, $^{3}J_{HH}$ = 8.4 Hz, $^{4}J_{HH}$ = 2.3 Hz, 2H, H_b), 7.46 (d, $^{3}J_{HH}$ = 8.5 Hz, 2H, H_c), 6.94 (s, 2H, H_d), 3.98 (t, $^{3}J_{HH}$ = 6.6 Hz, 2H, H_f), 2.58 (s, 6H, H_e), 1.79 (dt, $^{3}J_{HH}$ = 14.5 Hz, $^{3}J_{HH}$ = 6.7 Hz, 2H, H_g), 1.48 (p, $^{3}J_{HH}$ = 7.2 Hz, 2H, H_h), 1.37-1.34 (m, 4H, H_i+H_j), 0.93-0.90 (m, 3H, H_k) ppm. **^{13}C{^{1}H} NMR** (125 MHz; $CDCl_3$): δ_C 163.3, 156.5, 151.7, 151.7, 149.0, 140.8, 136.2, 136.0, 118.8, 110.3, 68.98, 31.6, 29.3, 25.7, 22.6, 15.6, 14.0 ppm. **MS**(MALDI): *m/z* 1270.8 $[M_2\text{-}Cl]^+$. **Anal. Calc.** for $C_{24}H_{27}ClN_2OPtS_2$: C, 44.07; H, 4.16; N, 4.28 %. **Found**: C, 43.67; H, 4.11; N, 4.00 %.

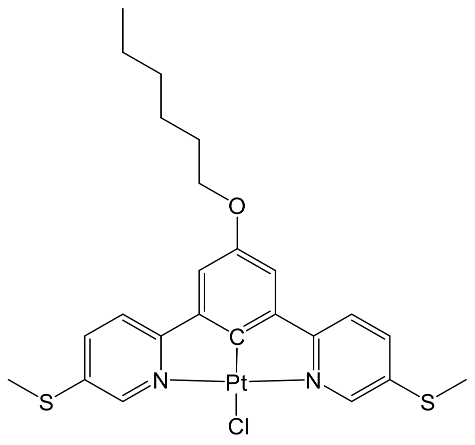

S2. NMR spectra of reported compounds

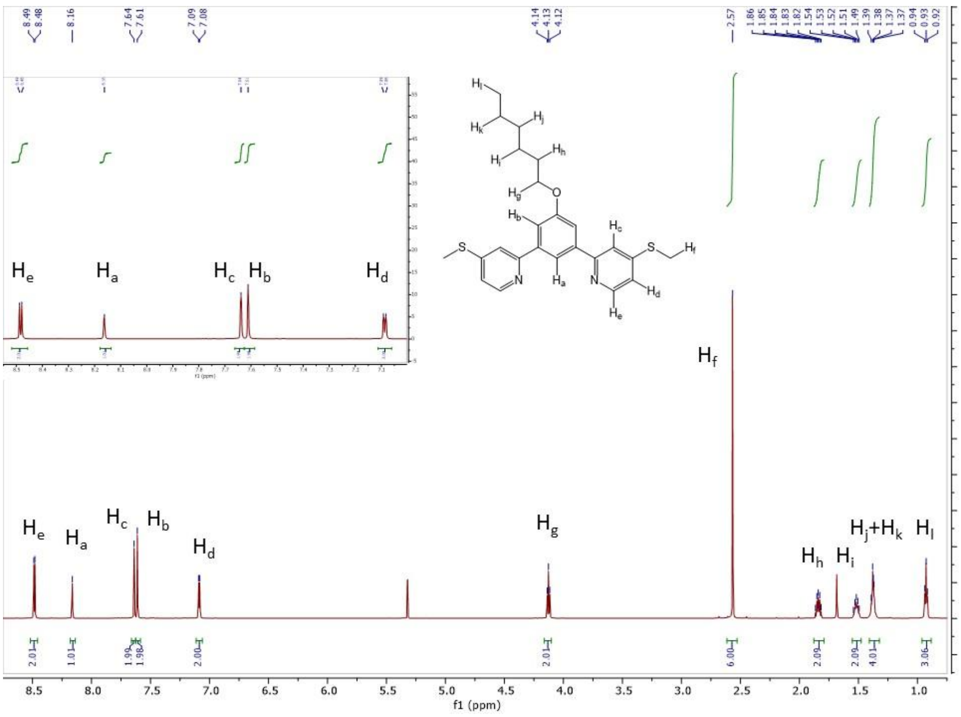

Figure S1. ^{1}H NMR spectrum of $\mathbf{L^m}$H recorded in CD_2Cl_2.

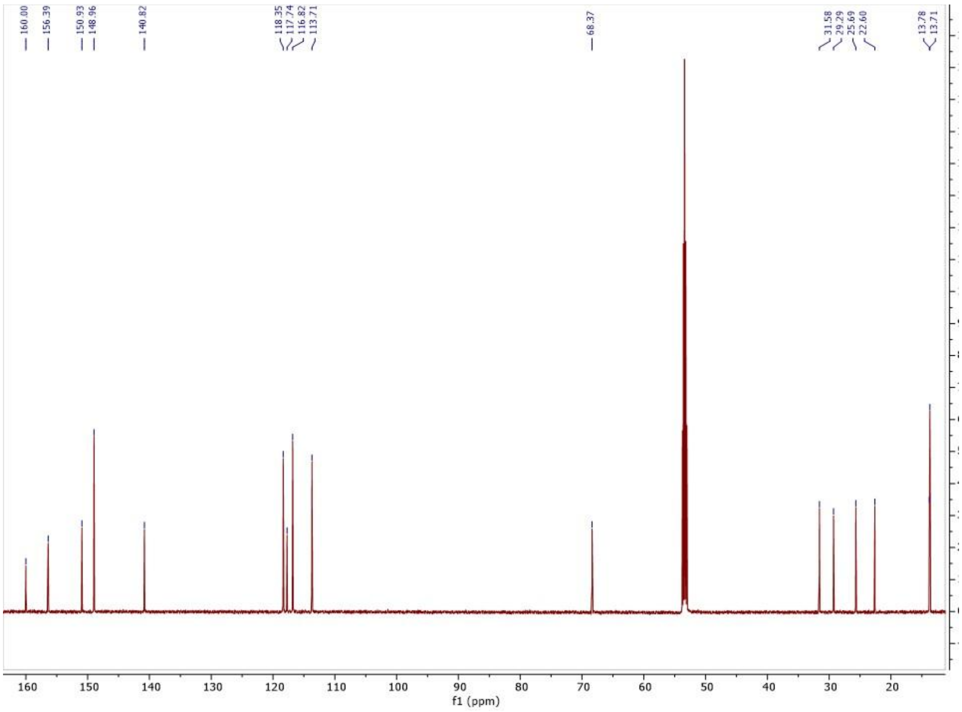

Figure S2. $^{13}C\{^{1}H\}$ NMR spectrum of $\mathbf{L^m}$H recorded in CD_2Cl_2.

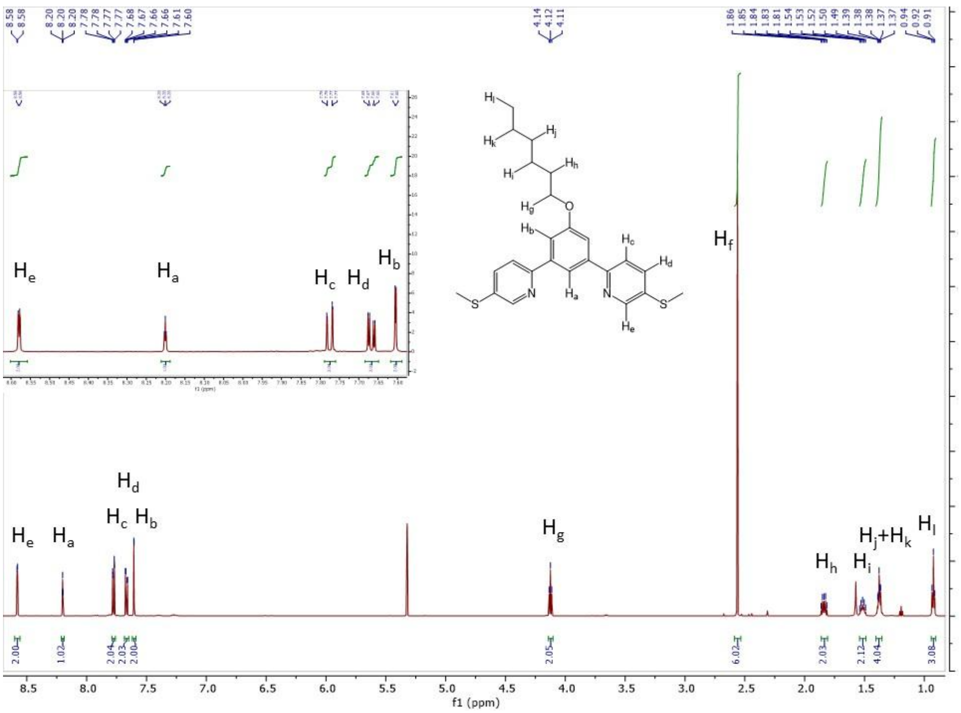

Figure S3. ^{1}H NMR spectrum of **L**^{p}H recorded in CD_2Cl_2.

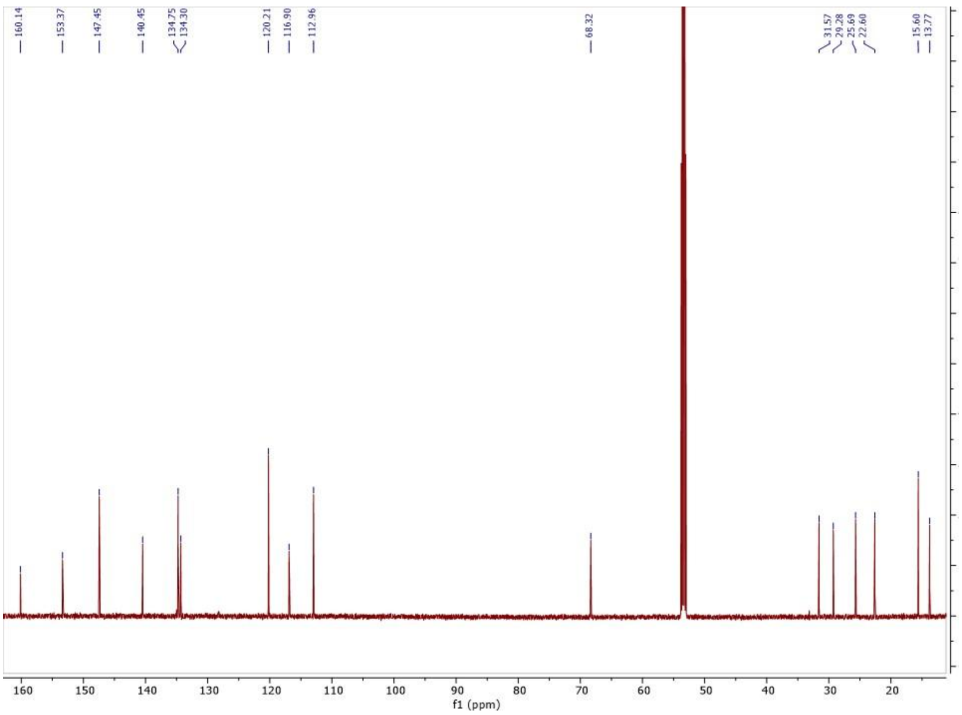

Figure S4. $^{13}C\{^{1}H\}$ NMR spectrum of **L**^{p}H recorded in CD_2Cl_2.

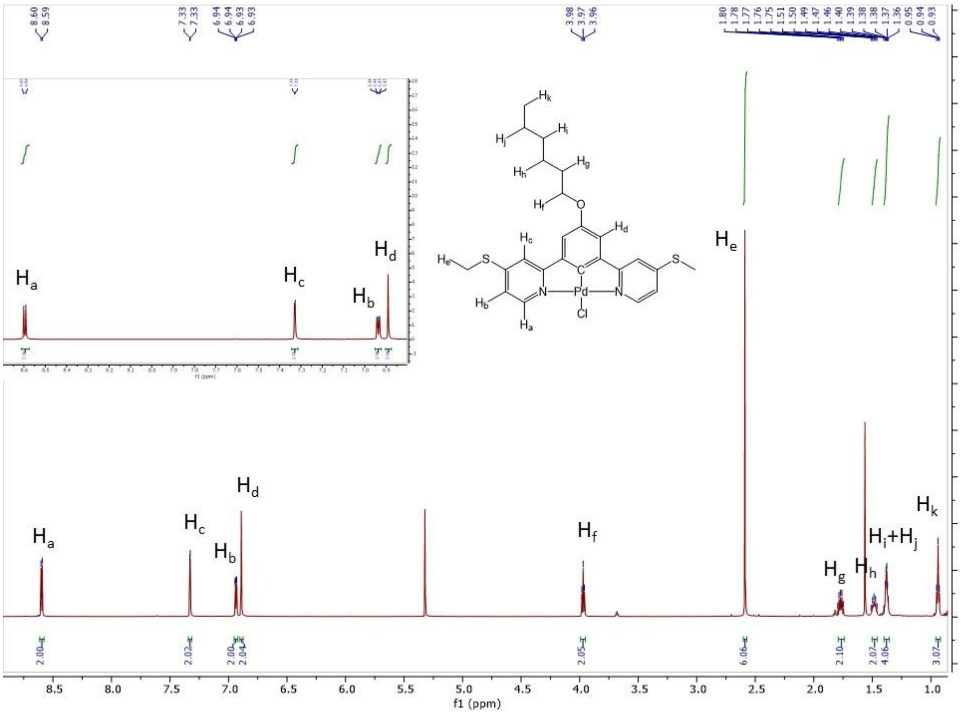

Figure S5. ^{1}H NMR spectrum of **Pd^m^** recorded in CD_2Cl_2.

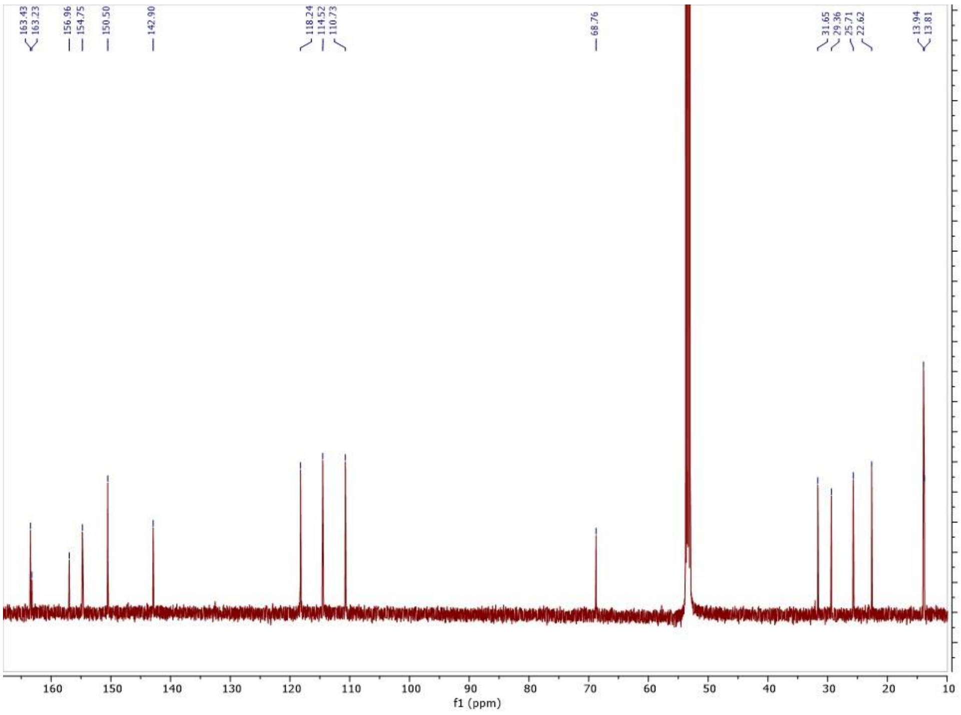

Figure S6. $^{13}C\{^{1}H\}$ NMR spectrum of **Pd^m^** recorded in CD_2Cl_2.

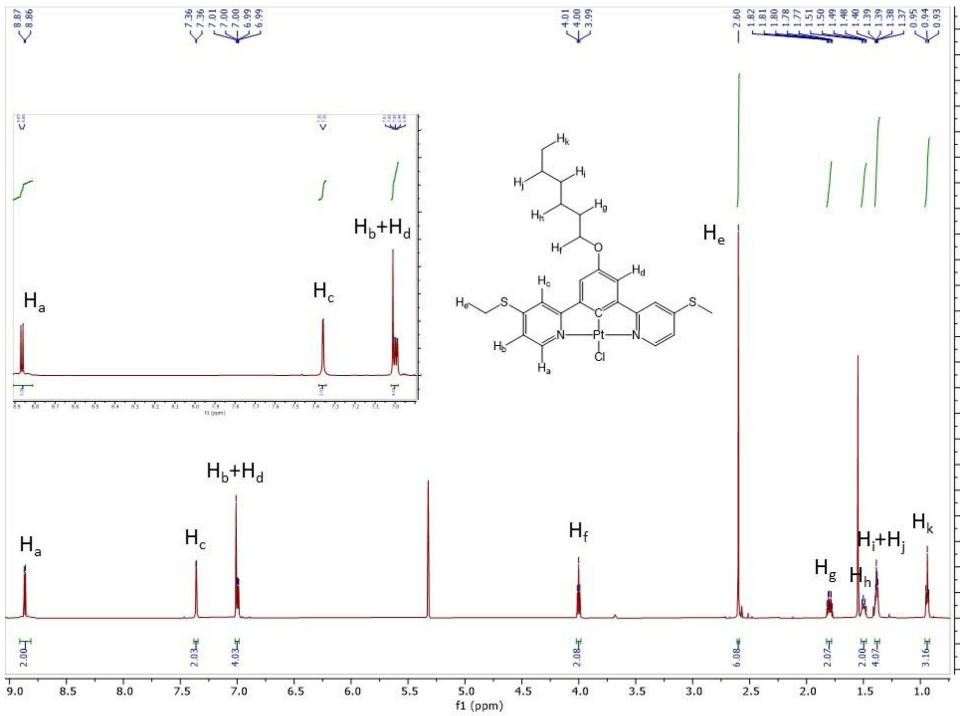

Figure S7. ^{1}H NMR spectrum of **Pt^m** recorded in CD_2Cl_2.

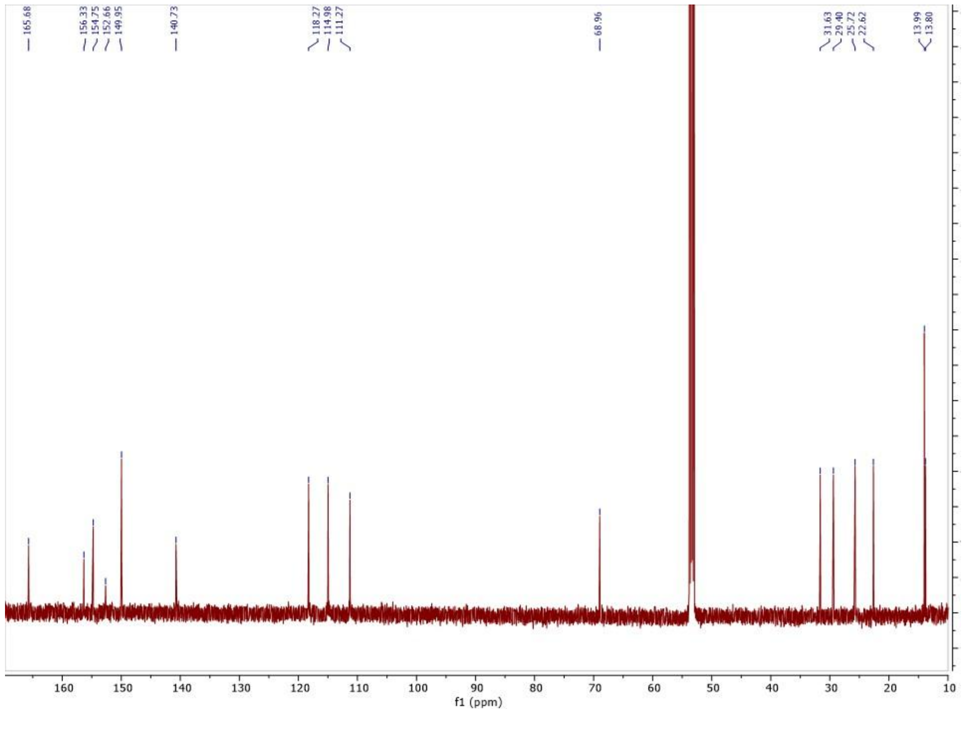

Figure S8. $^{13}C\{^1H\}$ NMR spectrum of **Pt^m** recorded in CD_2Cl_2.

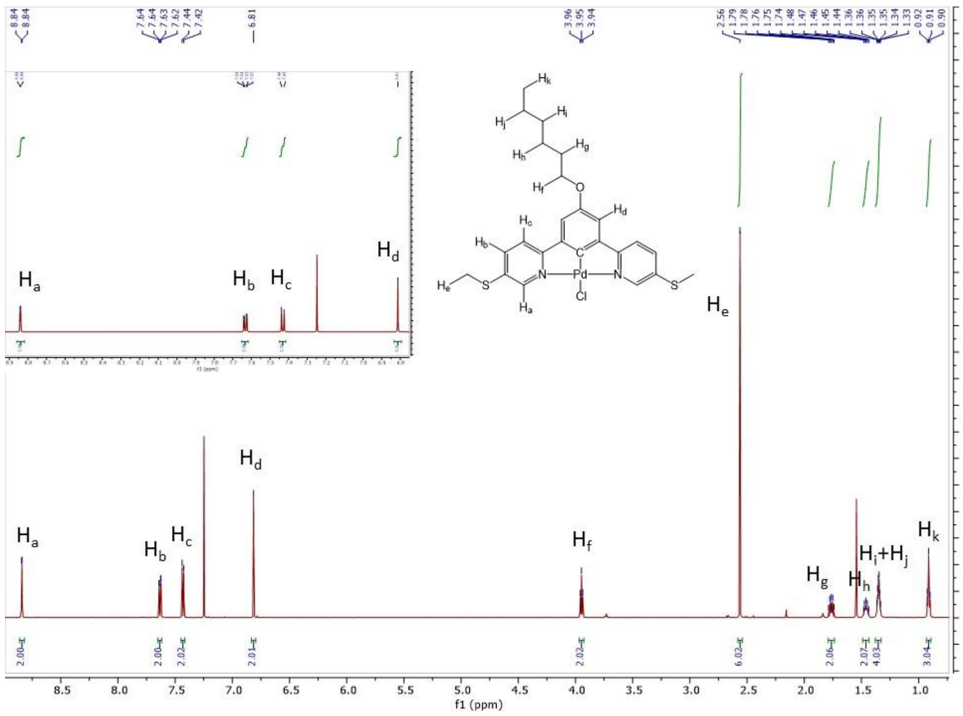

Figure S9. ^{1}H NMR spectrum of **Pd**^{p} recorded in CDCl$_3$.

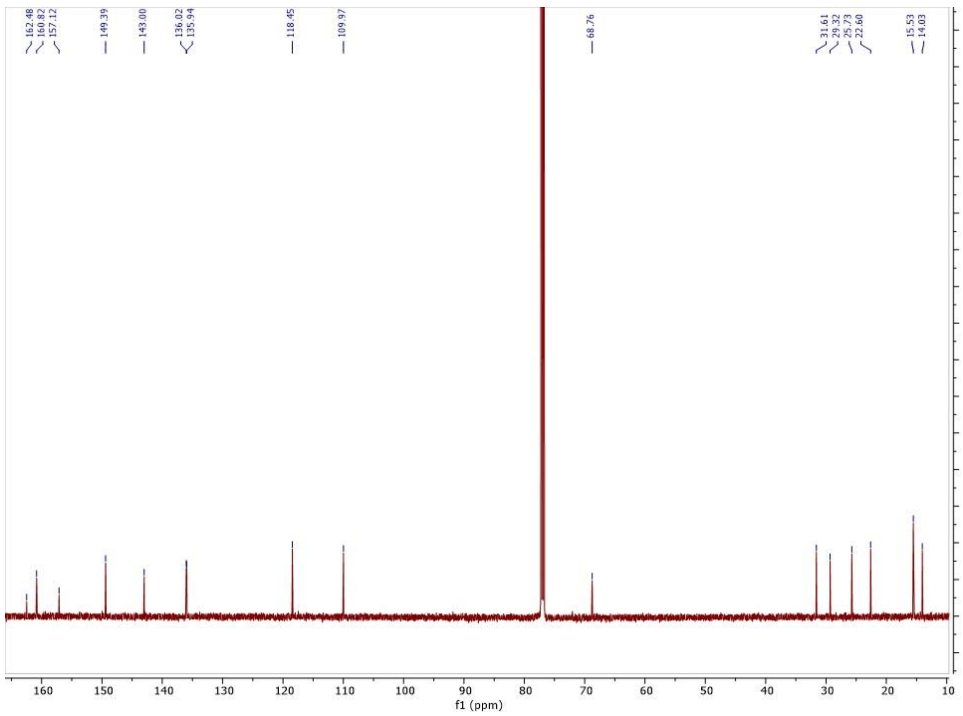

Figure S10. ^{13}C{^{1}H} NMR spectrum of **Pd**^{p} recorded in CDCl$_3$.

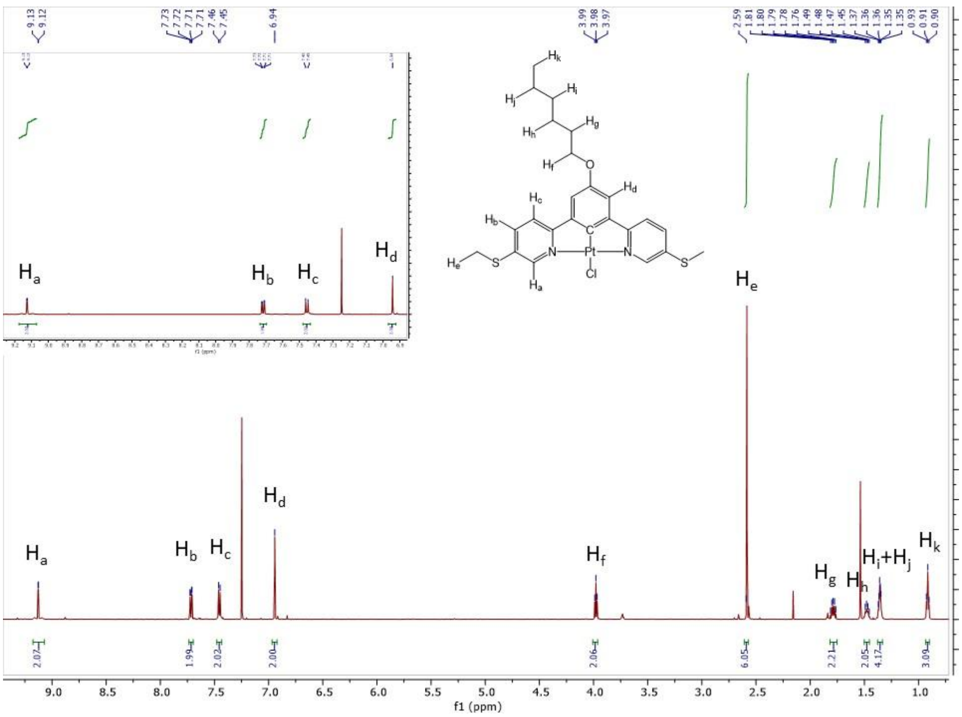

Figure S11. ^{1}H NMR spectrum of **Pt^p** recorded in $CDCl_3$.

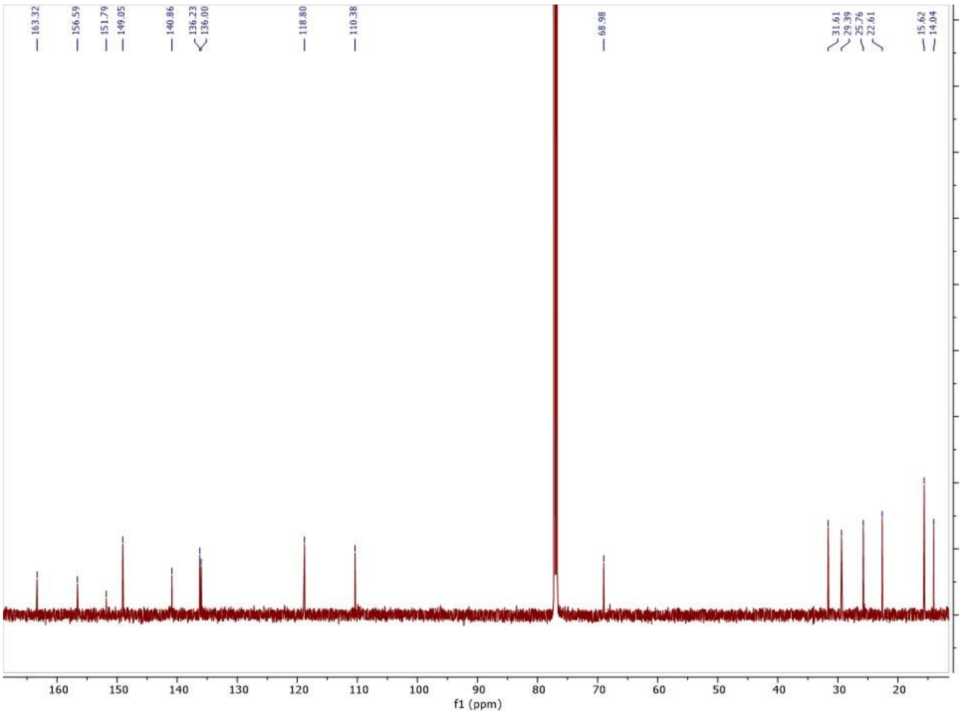

Figure S12. $^{13}C\{^1H\}$ NMR spectrum of **Pt^p** recorded in $CDCl_3$.

S3. X-ray Crystallography

Single-crystal X-ray diffraction experiments for **Pd^{m}** and **Pt^{m}** were carried out on a Bruker D8 Venture 3-circle diffractometer, equipped with a CPA area detector PHOTON III C14 MM, using Mo-$K\alpha$ radiation from Incoatec IµS 3.0 microsources with focusing mirrors. The crystals were cooled using a Cryostream 700 open-flow N_2 gas cryostat (Oxford Cryosystems). The data were collected in shutterless mode by narrow frame ω scans covering full sphere of reciprocal space, using APEX3 v.2017.3-0 software, reflection intensities integrated using SAINT v8.40A software (Bruker AXS, 2019). Data were corrected for absorption by semi-empirical methods based on Laue equivalents and multiple scans using SADABS 2016/2 software.[3] Both structures were solved by dual-space intrinsic phasing, using SHELXT 2018/2 program[4] and refined by full-matrix least squares using SHELXL 2018/3 software[5] on Olex2 platform.[6] Crystal data and other experimental details are listed in Table S1, molecular structures are shown in Figures S13-S15. In both structures the molecule has crystallographic inversion symmetry.

Table S1. Crystal data and structure refinement for structures **Pd^m** and **Pt^m**

	**Pd^m**	**Pt^m**
CCDC dep. number	2334297	2334298
Empirical formula	$C_{24}H_{27}ClN_2OPdS_2$	$C_{24}H_{27}ClN_2OPtS_2$
Formula weight	565.44	654.13
Temperature/K	120	120
Crystal system	monoclinic	monoclinic
Space group	$P2_1/n$ (no. 14)	$P2_1/n$ (no. 14)
a/Å	14.2242(6)	11.4228(4)
b/Å	11.2449(4)	28.9476(11)
c/Å	14.8315(6)	14.3008(6)
β/°	103.0865(14)	100.3393(15)
Volume/Å^3	2310.68(16)	4652.0(3)
Z	4	8
d_{calc}, g/cm^3	1.625	1.868
μ/mm^{-1}	1.119	6.347
F(000)	1152	2560
λ, Å	0.71073	0.71073
2 Θ_{max} /°	66	70
Reflections collected	60395	117750
independent	8712	19662
with $I \geq 2\sigma(I)$	7573	16910
R_{int}	0.049	0.042
Refined parameters	286	566
Goodness-of-fit on F^2	1.182	1.275
R_1 [$I \geq 2\sigma(I)$]	0.047	0.045
wR_2 [all data]	0.099	0.083
Residual $\Delta\rho_{max/min}$ / e Å^{-3}	1.91/-1.42	2.23/-1.98

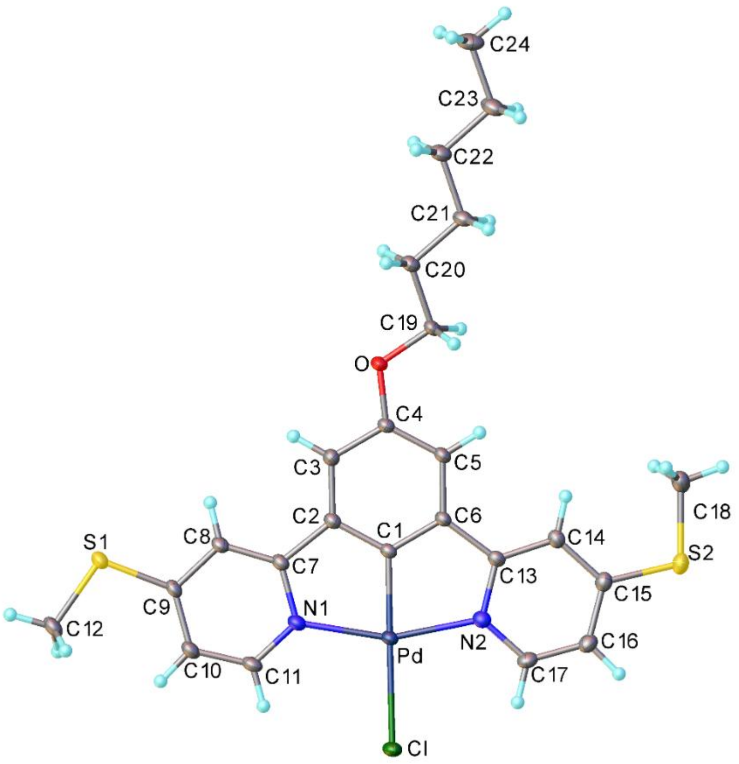

($\mathbf{Pd^{m}}$)

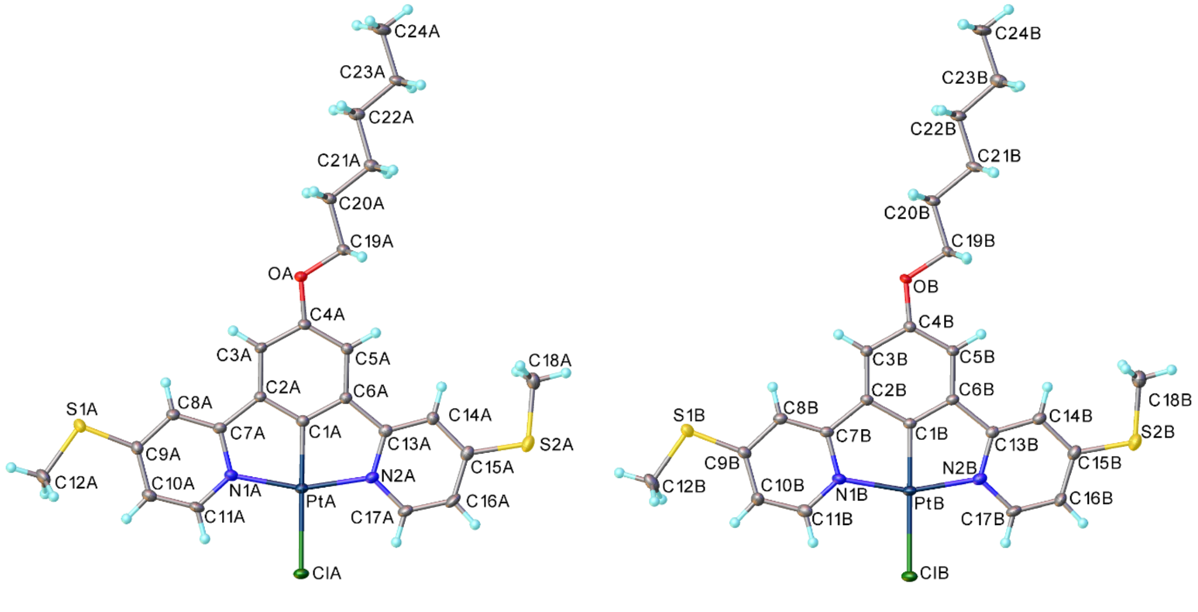

($\mathbf{Pt^{m}}$)

Figure S13. X-ray molecular structures of $\mathbf{Pd^{m}}$ and two symmetrically independent molecules of $\mathbf{Pt^{m}}$(A and B). Thermal ellipsoids are drawn at the 50% probability level.

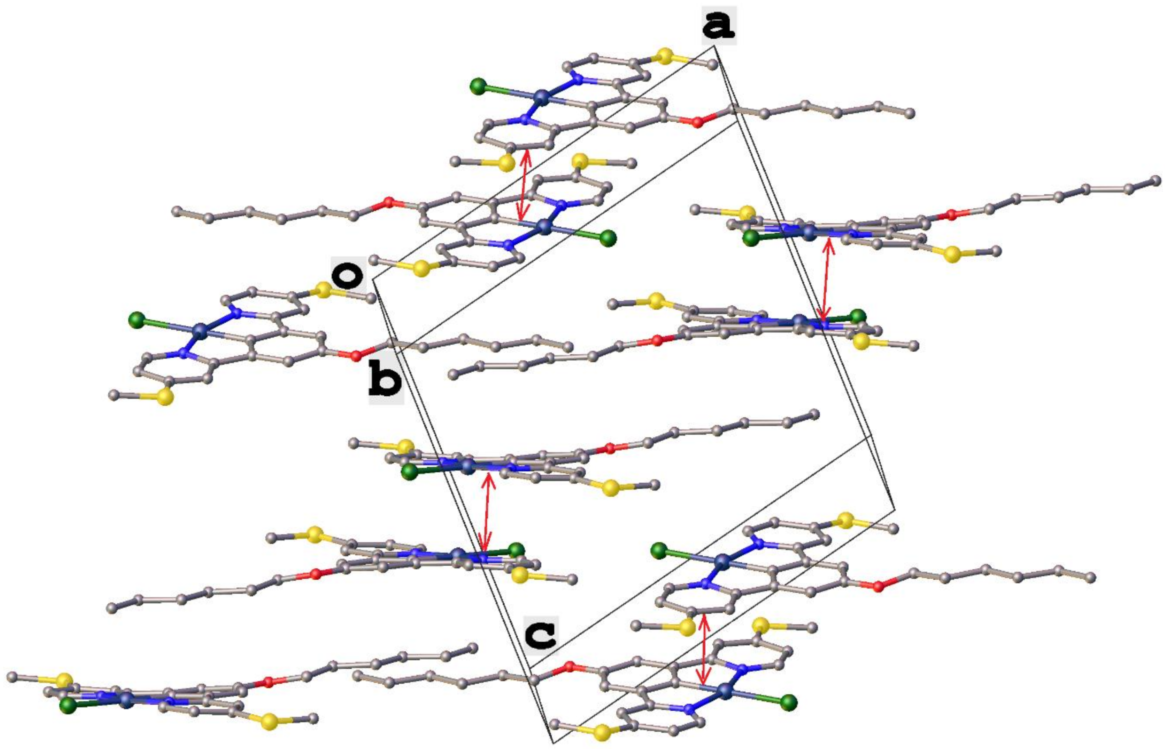

(a)

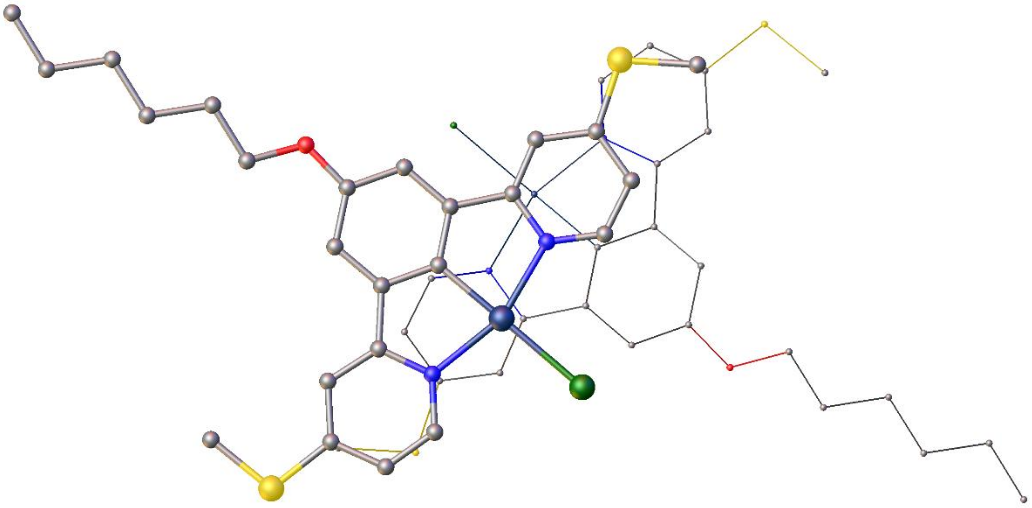

(b)

Figure S14. (a) Crystal packing of **Pd^{m}**; red arrows show π-π stacking; (b) overlap of stacked molecules (interplanar separation 3.38 Å). Hydrogen atoms are omitted for clarity.

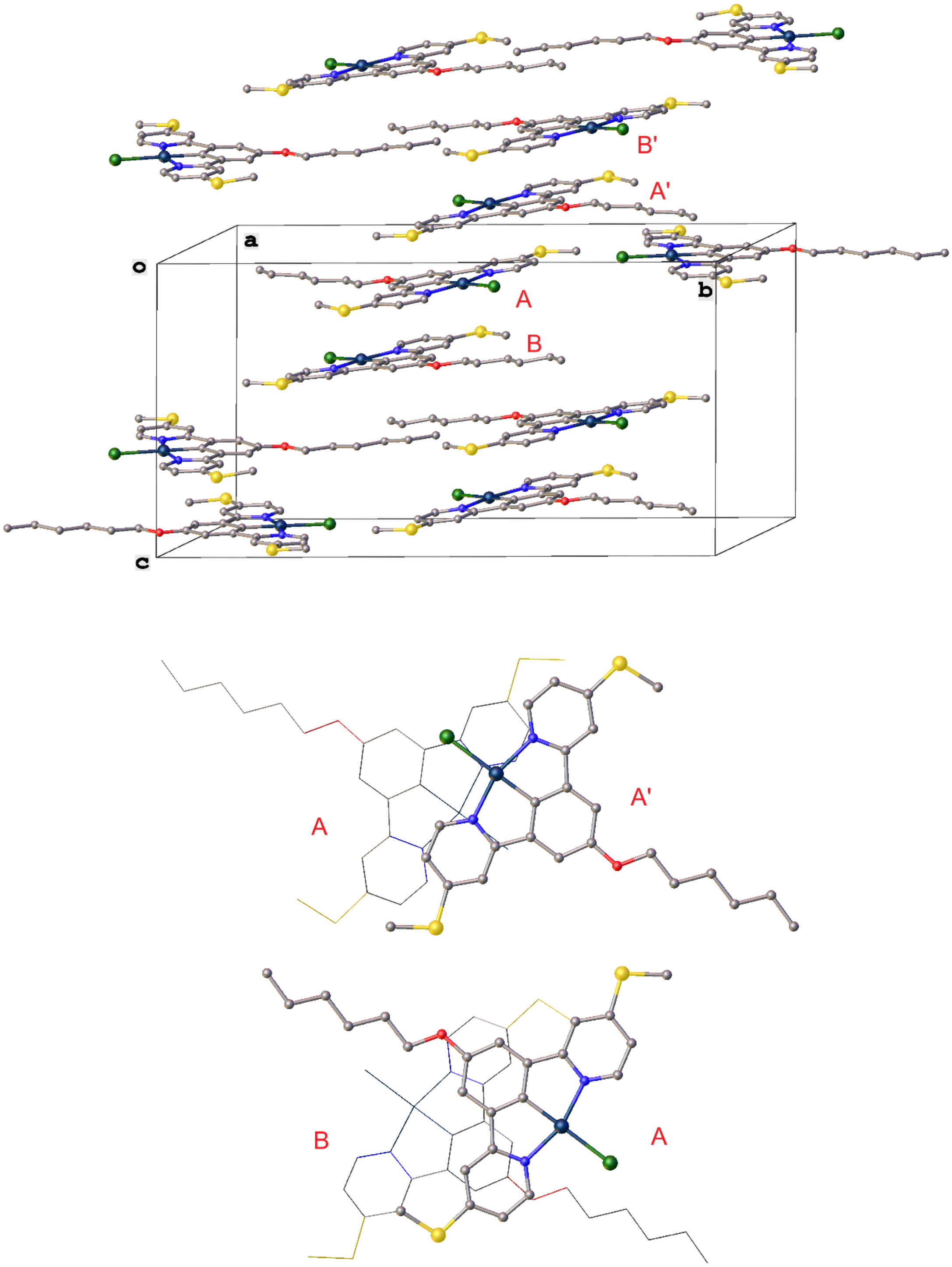

(a)

(b)

Figure S15. (a) Crystal packing of **Pt^m^**; primed molecules are generated by inversion (1-x, 1-y, -z); (b) overlap of π-π stacked molecules. Interplanar separations A...A' 3.42 Å (parallel planes), A...B 3.36 Å (interplanar angle 1.7°). Hydrogen atoms are omitted for clarity.

Table S2. Bond lengths (Å) in **Pd**^{m} and **Pt**^{m}

**Bond**	**Pd**^{m}	**Pt**^{m} **(A)**	**Pt**^{m} **(B)**
**M—C(1)**	1.911(2)	1.913(4)	1.917(3)
**M—N(1)**	2.065(2)	2.036(3)	2.036(3)
**M—N(2)**	2.062(2)	2.028(3)	2.027(3)
**M—Cl**	2.4440(6)	2.4306(9)	2.4394(9)

S4. Photophysical data for reported compounds

Instrumentation: All photophysical data were collected using DCM solutions of the respective compound. The UV-visible spectra were measured on a Unicam UV2-100 spectrometer operated with Unicam Vision software in quartz cuvettes with a path length l = 1 cm. Excitation and emission photoluminescence spectra were recorded on a Horiba Jobin Yvon SPEX Fluorolog 3-22 spectrofluorometer. Samples were degassed by repeated freeze-pump-thaw cycles using a turbomolecular pump until the pressure was stable in quartz cuvettes (l = 1 cm). The solutions had absorbance below 0.15 to minimize inner filter effects. Photoluminescence quantum yields (PLQYs) were measured following our previously reported method (see below).[7] Quinine sulfate in 0.1 M H_2SO_4 (Φ_F: 0.546)[8] was used as the reference and the emission spectra of quinine sulfate were collected by exciting the samples at 360 nm.

Emission lifetimes were determined using a custom spectrometer measured by time-correlated single photon counting (TCSPC) using a pulsed diode laser (371 nm; IBH Ltd) running at 1 MHz. The fluorescence emission was collected at right angles to the excitation source. The emission wavelength was selected using a Horiba Jobin Yvon Triax 190 monochromator and detected by a cooled IBH TBX-04 PMT. Timing was achieved using an Ortec 567 time-to-amplitude converter and an E. G. & G Trumpcard pulse height analyzer (PHA), and data was recorded using Maestro (version 510) software. The data were transferred to a PC and analyzed using non-linear regression to a single exponential decay, and the quality of fit was established by reduced χ^2 and random residuals. The decay data were fitted to a single exponential function.

The HOMO-LUMO gap, ΔE(H-L), for each compound was determined from their electronic absorbance spectra (see Figure S16, Table S3). From this, we can establish that the

coordination of a metal ion to the ligand results in a significant reduction of the H-L energy gap. Moreover, each *para* compound had a lower H-L energy gap than their *meta* analogs which is attributed to the slight conjugation extension resulting from the position of the thiomethyl groups.

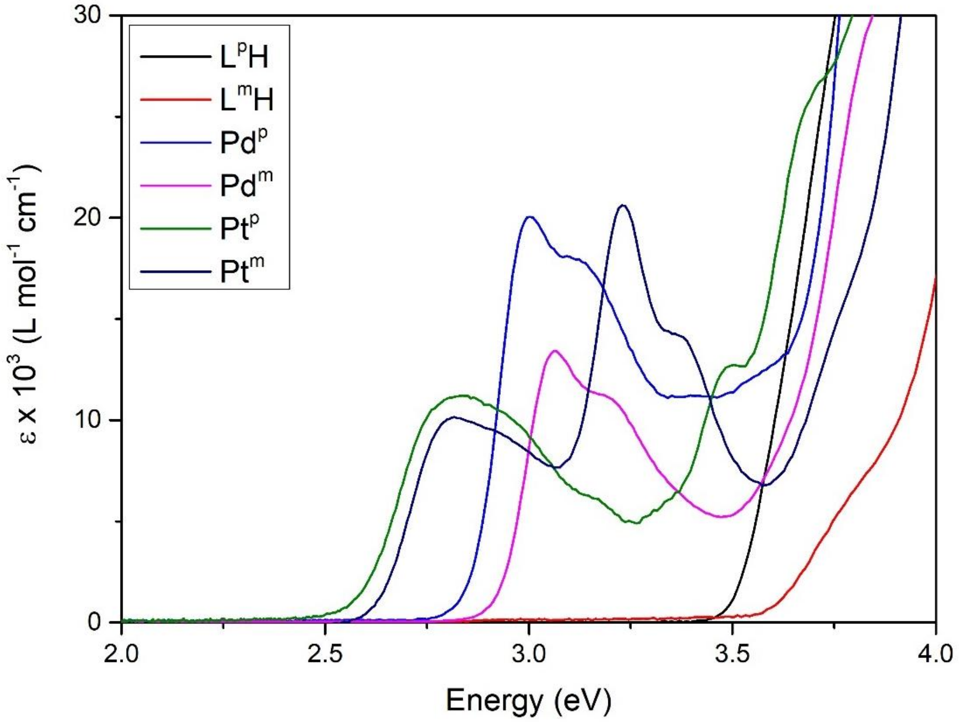

Figure S16. Electronic absorbance spectra of the measured compounds recorded in DCM.

Table S3. Δ*E*(H-L) determined for each compound based on their absorbance spectra.

Compound	Δ*E*(H-L) (eV)
L^pH	3.52
L^mH	3.58
**Pd^p**	2.84
**Pd^m**	2.91
**Pt^p**	2.58
**Pt^m**	2.62

As typically observed with platinum complexes of this nature,[8-11] both **Pt^m** and **Pt^p** were highly emissive. Similarly to the absorbance data, **Pt^p** displays a red shift of emission of *ca*. 75 meV relative to **Pt^m** consistent with a slightly extended conjugation associated with the *para*-position of the thiomethyl group (see Figure S17).

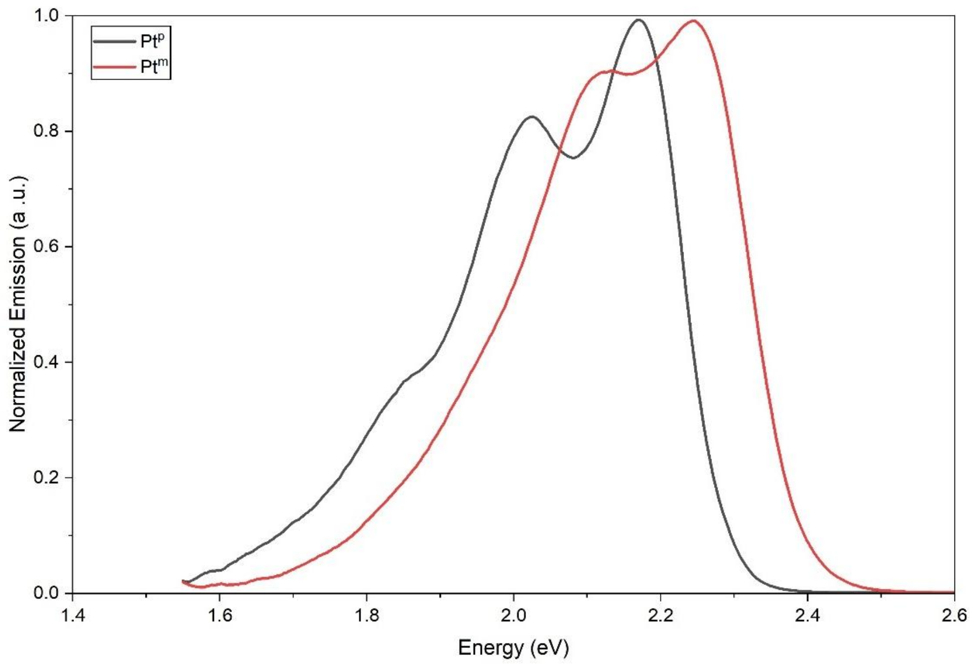

Figure S17. Emission spectra of **Pt^m** and **Pt^p** recorded in DCM, λ_{ex} = 450 nm.

Table S4. Photophysical data for $\mathbf{Pt^m}$ and $\mathbf{Pt^p}$.

Compound	Lifetime (τ, μs)	k_r (10^4 s^{-1})	k_{nr} (10^5 s^{-1})	PLQY Φ	Pure radiative lifetime (τ_0, μs)
$\mathbf{Pt^p}$	6.65	2.11	1.29	0.14	47.4
$\mathbf{Pt^m}$	7.00	12.7	0.15	0.89	787

S5. STM-BJ measurements

Conductance histograms

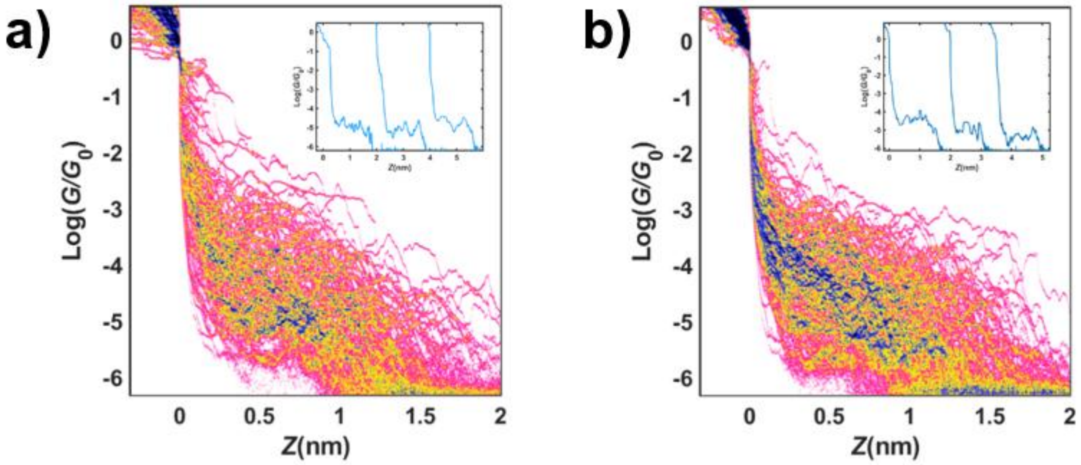

Figure S18. 2D conductance vs distance histograms for compounds (a) **L**^{m}H and (b) **L**^{p}H measured in air conditions; (inset) examples of individual traces.

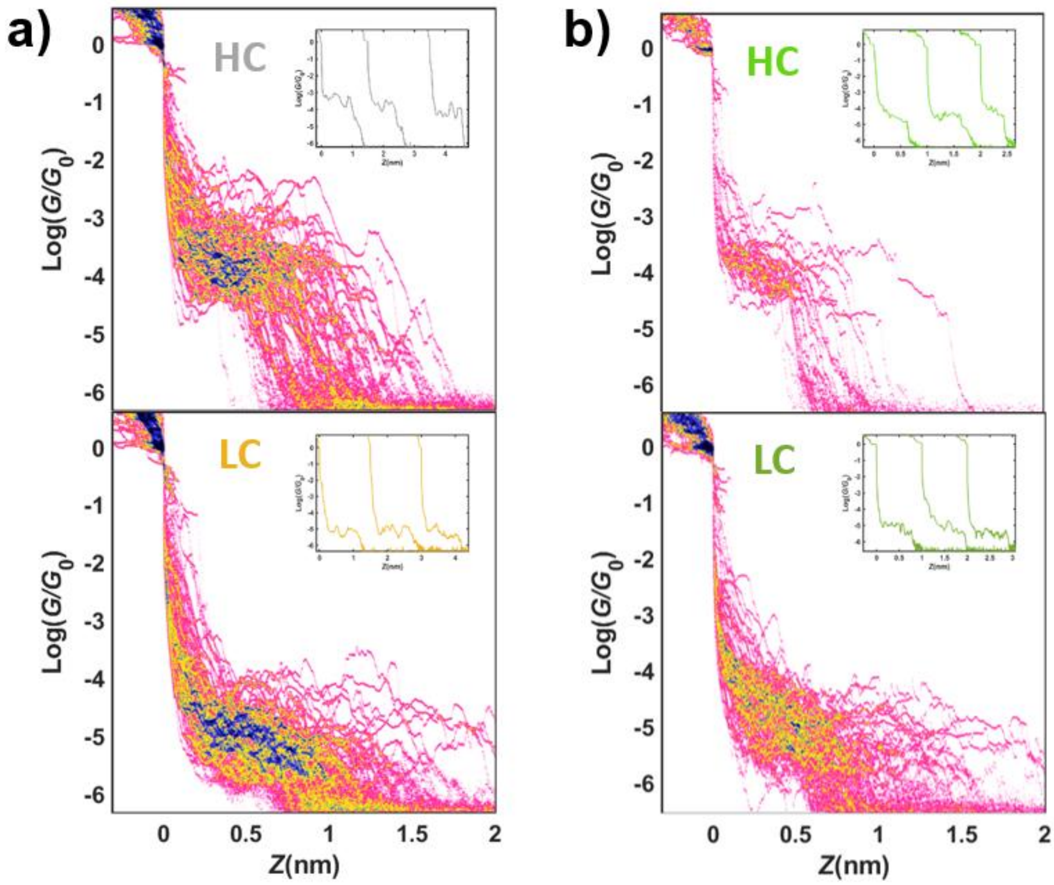

Figure S19. 2D conductance vs distance histograms of HC and LC features for compounds (a) **Pd^m** and (b) **Pd^p** measured in air conditions; (inset) examples of individual traces.

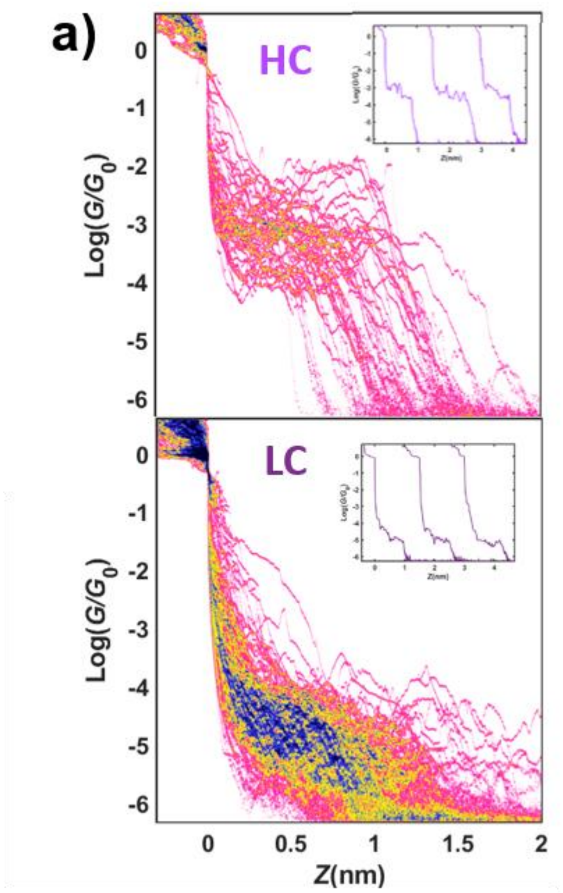

Figure S20. 2D conductance vs distance histograms of HC and LC features for compounds (a) **Pt^{m}** and (b) **Pt^{p}** measured in air conditions; (inset) examples of individual traces.

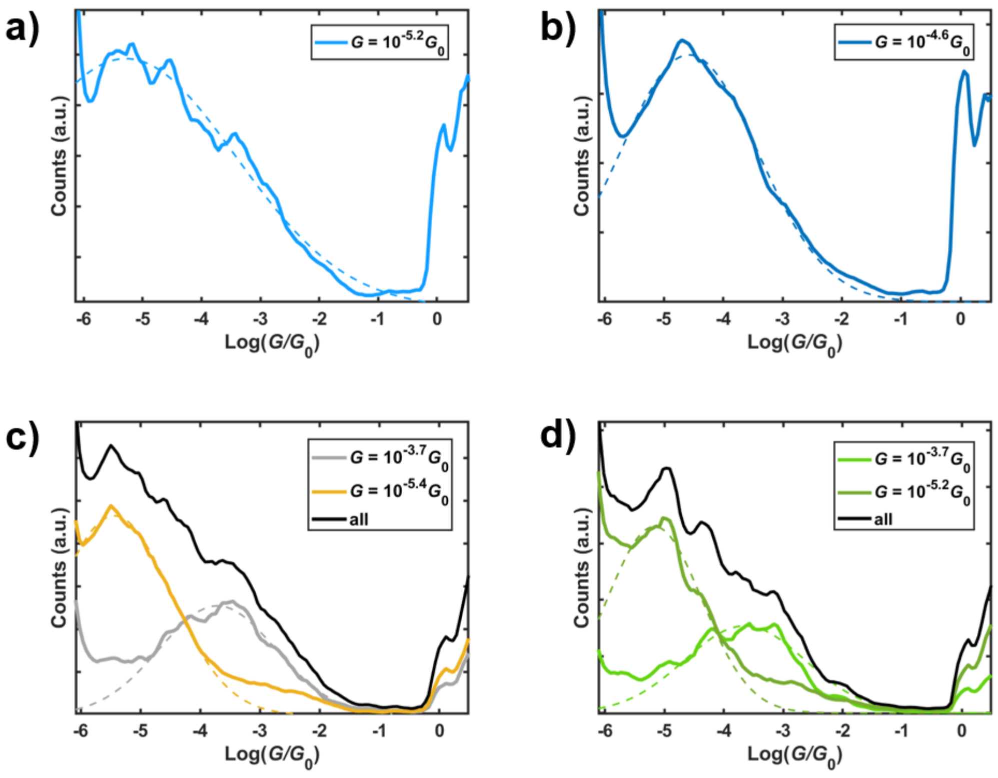

Figure S21. 1D conductance histograms for compounds (a) **L^m**H, (b) **L^p**H, (c) **Pd^m** and (d) **Pd^p** measured in solution. For each of the complexes two classes are assigned, HC and LC peaks. Gaussian fitted dashed lines are given to highlight the conductance peaks with conductance values being determined from their mean value, given in the respective legend.

Table S5. The relative percentage of the selected traces showing molecular features contributing to the High Conductance (HC) class / Low Conductance (LC) class for all data sets. The rows correspond to the measurements performed on a sample dry with the molecules deposited on top, and in solution.

Molecule	L^{m}H	L^{p}H	Pd^m	Pd^p	Pt^m	Pt^p
**Dry (%)**	100	100	56.0 / 44.0	25.4 / 74.6	22.4 / 77.6	10.1 / 89.9
**Solution (%)**	100	100	43.6 / 56.4	30.1 / 69.9	43.2 / 56.8	43.5 / 56.5

Concentration dependence

To further elucidate the origin of each conductance class, **Pt^{p}** was deposited on the gold substrate at concentrations of 2.0, 1.0, and 0.5 mM in DCM (see Figure S22). By lowering the concentration of molecules on the surface, the probability of intermolecular interactions in the junctions will decrease, thereby increasing the distance between each molecule on the surface.

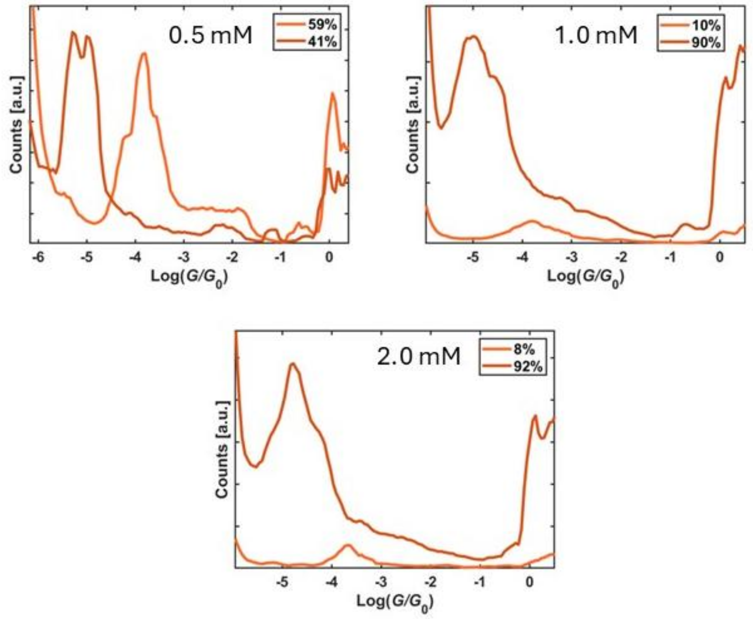

Figure S22. 1D conductance histograms for **Pt^{p}** recorded at 0.5, 1.0, and 2.0 mM. For each concentration, two classes are assigned: HC (orange) and LC (red) peaks.

Figure S22 shows that at 0.5 mM, the HC feature at G = $10^{-3.8}$ G_0 accounts for 59% of the traces, while at 1.0 – 2.0 mM this class accounts for only 10 and 8%, respectively, while the LC feature at G = $10^{-4.9}$ G_0 shows an inverse relation. From this information, we can attribute the LC to an intermolecular interaction, while the HC feature is due to a monomolecular junction. Although the measurements at 0.5 mM showed greater resolution between classes, the junction-formation hit rate was only 0.24%, while the 1.0 mM measurements had a hit rate of 10%. To collect a sufficient number of successful molecular junctions, we therefore favored collecting data at a concentration of 1.0 mM rather than at a lower concentration.

S6. Junction length determination

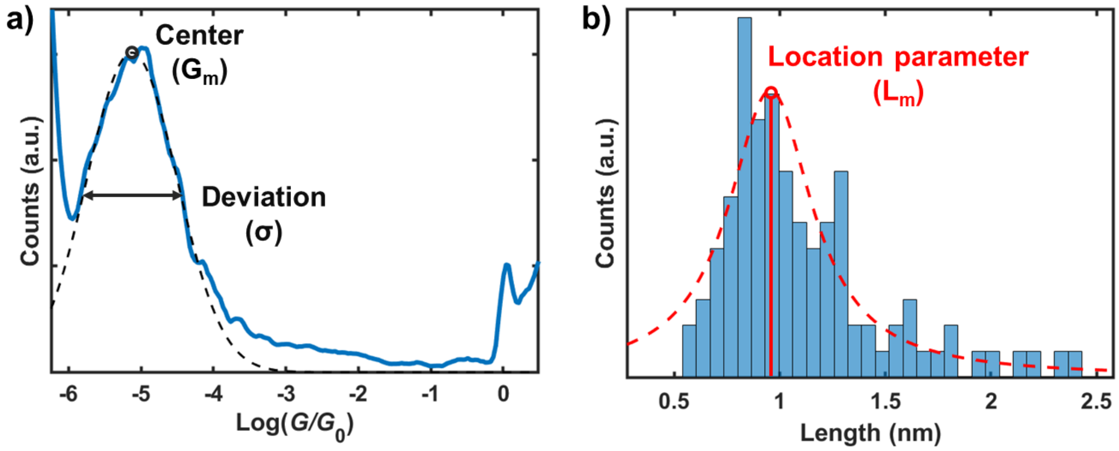

Figure S23. (a) Gaussian fitting of the 1D conductance histogram of compound **Pd^m**. (b) Length histogram obtained with the counts as the points with higher distance value within $G_m \pm 2\sigma$ for each trace. Lorentzian fitting as a dashed line, where the location parameter (L_m) is the mean length value.

Table S6. Summary of SCXRD and DFT calculated intramolecular S…S distances (nm).

Molecule	**L^{m}H**	**L^{p}H**	**Pd^m**	**Pd^p**	**Pt^m**	**Pt^p**
SCXRD			1.273		1.275	
DFT	0.787 – 1.267	1.226	1.281	1.101	1.279	1.049

S7. XPS Results

Binding energies were calibrated according to the C1s peak at 284.6 eV. The C1s peak was chosen, rather than for example Au4f, for consistency with all the samples (powders and SAMs) although possible inaccuracies of using the C1s binding energy have been reviewed.[12] XPS peak fitting was carried out using the CASA software with the Shirley BG type.[13]

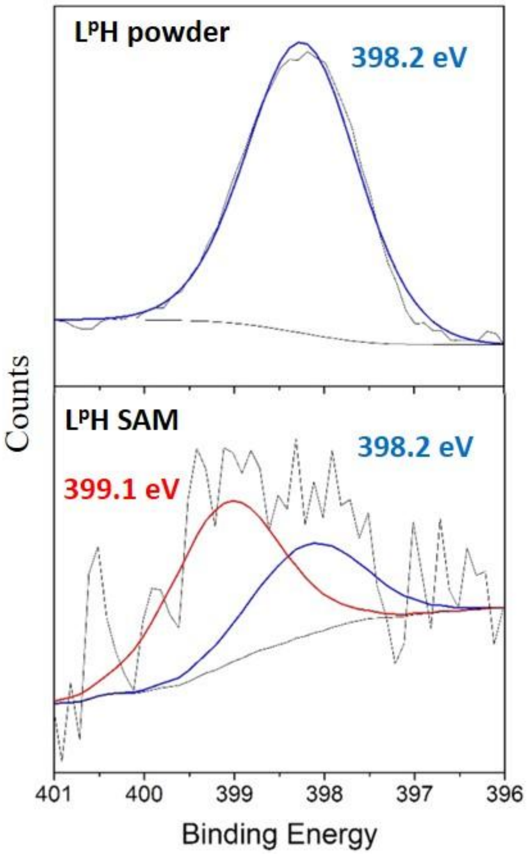

Figure S24. XPS spectra in the N1s region corresponding to **L**ᴾH powder and SAM.

S8. Thermopower measurements

The Seebeck coefficients (*S*) of **Pd^m** and **Pd^p** were measured using the method described by Evangeli et al.[14] to provide insight into the nature of the molecules' conductance behavior, i.e. is it HOMO- or LUMO-dominated? The measurements focused on the HC feature at its higher conductance improved precision and corresponded to the S…S contacted junction. This gave S = -3.1 μV/K (**Pd^m**) and S = -1.2 μV/K (**Pd^p**) (Figure S25); the negative values are consistent with LUMO-dominated behavior. The *meta* positions of the thiomethyl anchors in **Pd^m** having a larger magnitude Seebeck coefficient than **Pd^p** in contrast to literature examples of linkage isomerization.[15, 16]

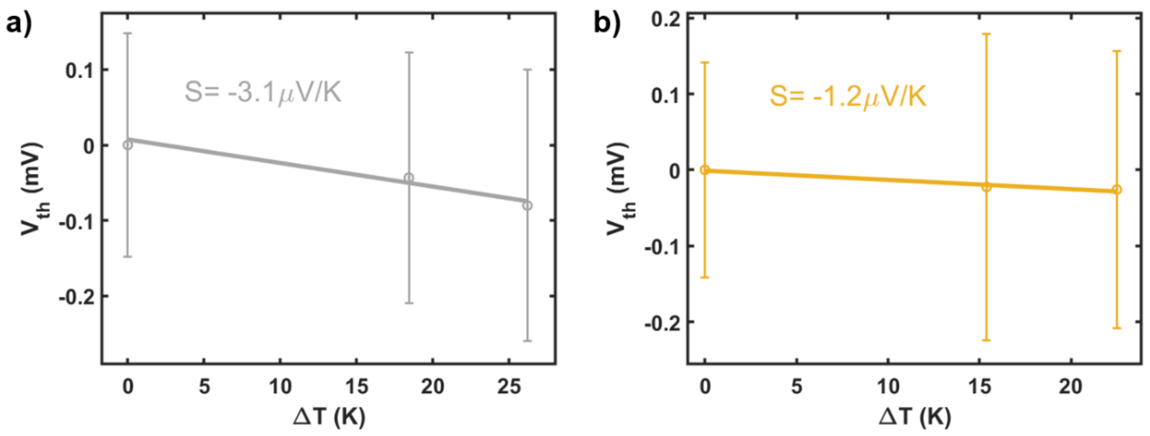

Figure S25. Temperature difference dependence of the thermovoltage for HC features of (a) **Pd^m** and (b) **Pd^p** compounds. The slope of the linear fitting of the data points is the Seebeck coefficient.

S9. Theory

For each of the compounds, two distinct conductance features were observed. Here we consider the possible explanations for this behavior of the ligands; it is possible that the pyridyl groups were also acting as contact groups in addition to the thiomethyl groups. Another possibility that could apply to each of the compounds is the formation of π-π stacked dimers. Here we will examine each in turn.

First, we consider the potential of the pyridine nitrogen lone pair acting as an anchor group. For this to occur, the pyridyl ring would have to rotate to a position that was accessible to the electrode. To examine this, the energy difference of this rotation was calculated by scanning the dihedral angle formed between the pyridyl and phenyl rings using B3LYP; for the ligands (**L**^{m}H and **L**^{p}H) a 6-31G(d) basis set was employed for all the atoms. Given that both pyridyl rings were capable of rotation, the first scan was performed with both rings in a comparable position to the complexes, while a second scan was performed with one of the pyridyl rings in the second stable position (180 ° from the original position). As shown in Figure S25, there were two preferred orientations associated with the pyridyl ring being planar with the rest of the ligand, even though the maximum energy difference between orientations was 0.22 eV, suggesting complete rotation is unlikely to occur at room temperature but rotations as large as 60 ° could readily occur. However, it should be noted that this rotation was only possible for the free ligands and would not explain the multiple conductance features in the metal complexes.

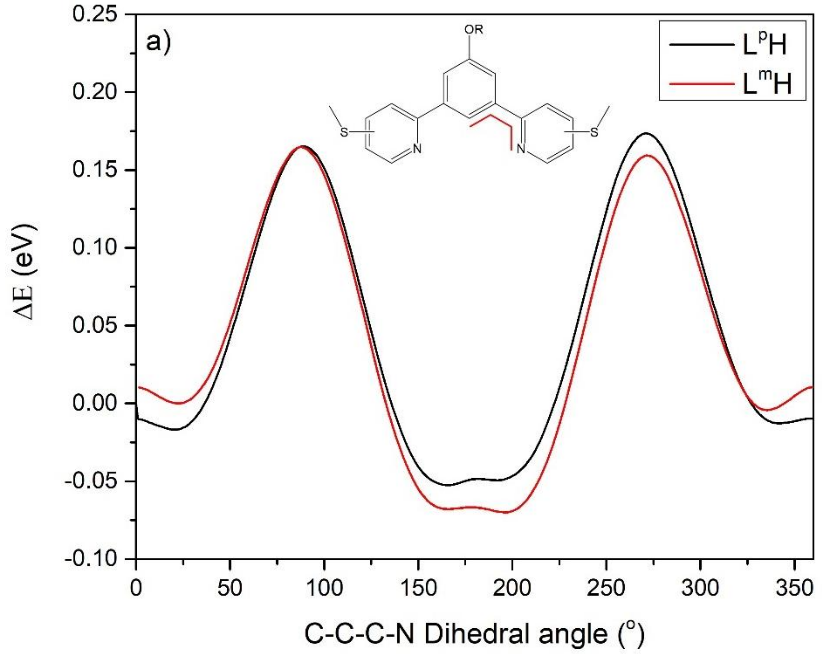

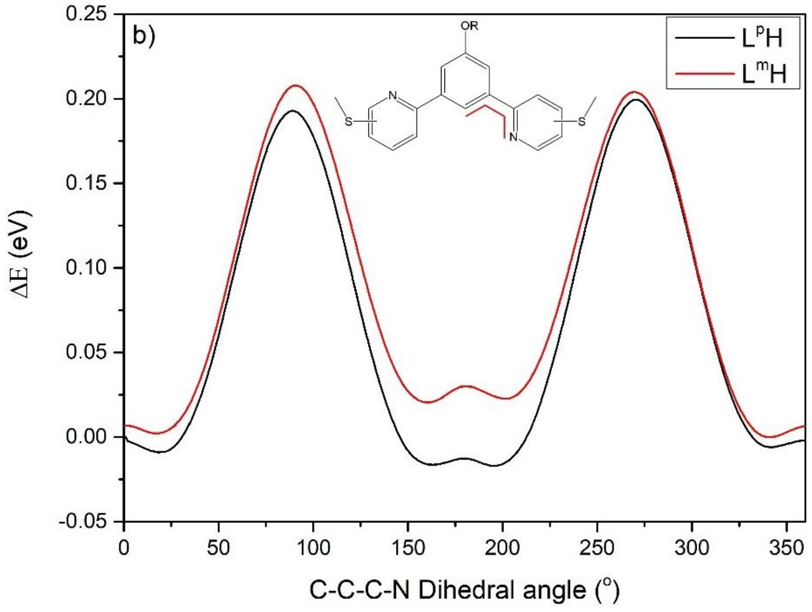

Figure S26. Energy difference (ΔE) associated with the rotation of the pyridyl rings about the CCCN dihedral angle: a) both pyridyl rings in a symmetrical starting position, b) asymmetric pyridyl rings starting in opposite positions.

The possibility of *π*-*π* stacking between the molecules was examined by comparing the energy difference between the molecules with and without π-stacking present. To achieve

this, the isolated molecules and the corresponding dimers were geometrically optimized using an LSDA functional and 6-31G(d) basis set for the ligands (**L**^{m}H and **L**^{p}H), while a 6-31G(d)/LANL2DZ basis set was employed for both the palladium (**Pd**^{m} and **Pd**^{p}) and platinum (**Pt**^{m} and **Pt**^{p}) complexes. To determine the relative energy difference due to the π-stacking, the energy of the isolated molecules (open) was used as E = 0.000 eV. Based on the geometry of the molecules, two possible orientations for the π-stacking (closed) were considered — the first 'asym' being when the molecules were stacked directly with only vertical translation, and the second 'sym' where the second molecule was stacked directly with both vertical translation and a 180 ° rotation. Figures S27–S29 show the relaxed structures of the isolated molecules and dimers with their corresponding differences. For each of the compounds π-stacking was energetically favoured in the gas phase, suggesting that this was likely to occur; however, there was not a clear energetic difference between either of the geometries, therefore it cannot be determined which orientation is favored in the junction.

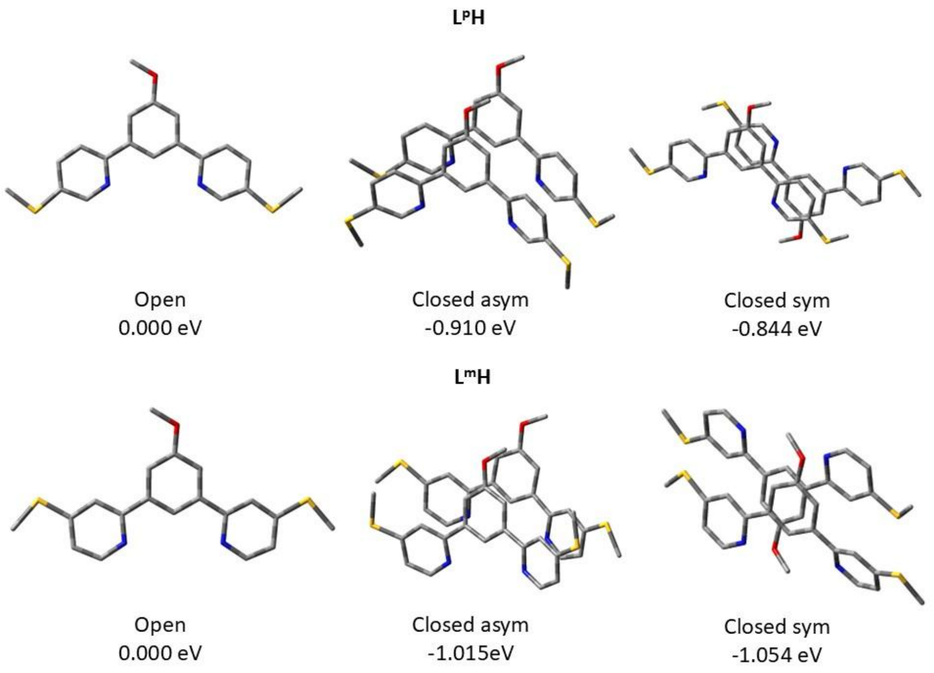

Figure S27. Geometries and energy differences for the isolated and π-π stacked ligands (**L**^{m}H and **L**^{p}H)

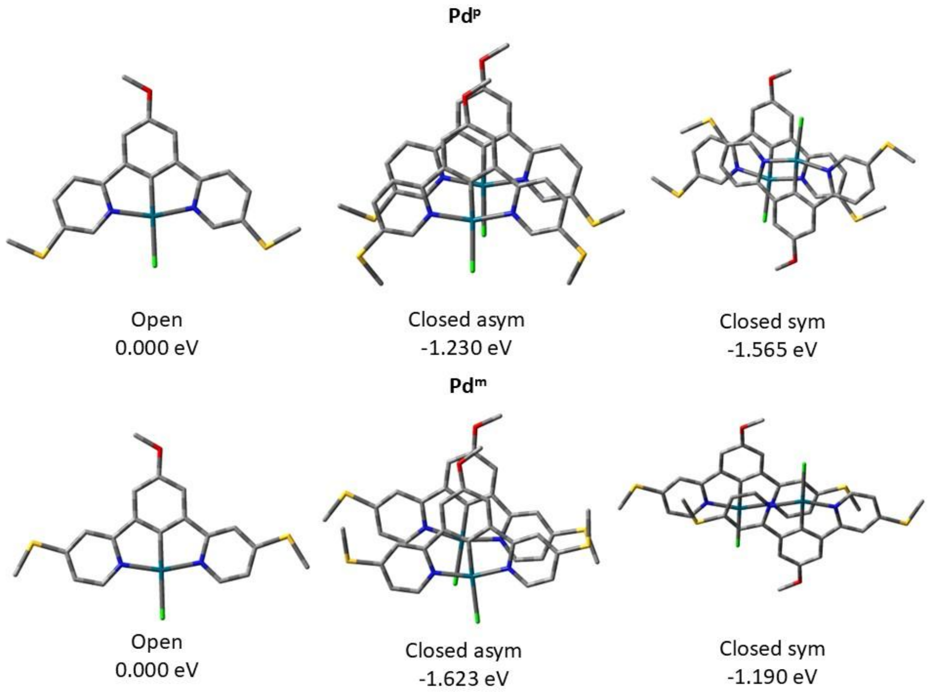

Figure S28. Geometries and energy differences for the isolated and π-π stacked palladium complexes (**Pd^{m}** and **Pd^{p}**)

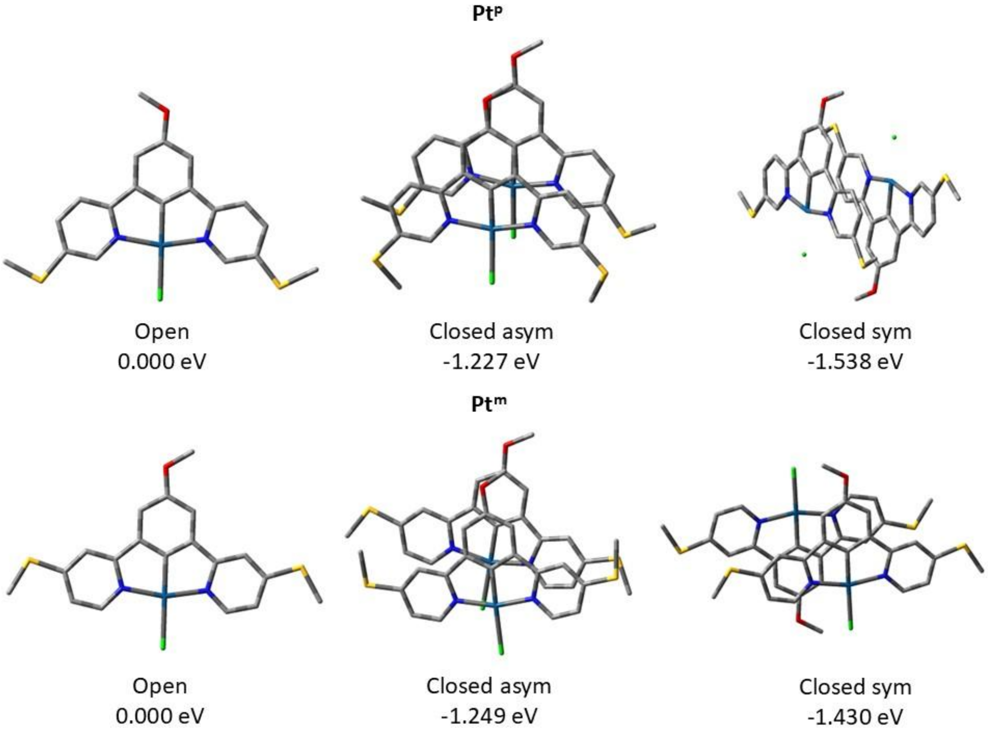

Figure S29. Geometries and energy differences for the isolated and π-π stacked platinum complexes (**Pt^{m}** and **Pt^{p}**)

Computational methods

The geometry of each structure studied in this paper was relaxed to the force tolerance of 10 meV/Å using the SIESTA[17] implementation of DFT, with a double-ζ polarized basis set (DZP) and the Generalized Gradient Approximation (GGA) functional with Perdew-Burke-Ernzerhof (PBE) parameterization. A real-space grid was defined with an equivalent energy cut-off of 250 Ry. We then calculated molecular orbitals of gas phase molecules.

To calculate electronic properties of the device, from the converged DFT calculation, the underlying spin polarized mean-field Hamiltonian H was combined with the quantum transport code, GOLLUM.[18, 19] This yields the transmission coefficient $T(E)$ for electrons of energy E (passing from the source to the drain) via the relation $T(E) = Tr(\Gamma_L(E)\mathrm{G}^{\mathrm{R}}(E)\Gamma_R(E)\mathrm{G}^{\mathrm{R}\dagger}(E))$ where $\Gamma_{L,R}(E) = i(\Sigma_{\mathrm{L,R}}(E) - \Sigma_{\mathrm{L,R}}{}^{\dagger}(E))$ describes the level broadening due to the coupling between left L and right R electrodes and the central scattering region, $\Sigma_{\mathrm{L,R}}(E)$ are the retarded self-energies associated with this coupling and $\mathrm{G}^{\mathrm{R}} = (ES - H - \Sigma_{\mathrm{L}} - \Sigma_{\mathrm{R}})^{-1}$ is the retarded Green's function, where H is the Hamiltonian and S is the overlap matrix obtained from SIESTA implementation of DFT. The electrical conductance is calculated using Landauer formula $G = G_0 \int_{-\infty}^{+\infty} dE\ T(E)(-\partial f(E,T,E_F)/\partial E)$, where $f = (e^{(E-E_F)/k_BT} + 1)^{-1}$ f is the Fermi-Dirac probability distribution function, T is the temperature, E_F is the Fermi energy, $G_0 = 2e^2/h$ is the conductance quantum, e is electron charge and h is the Planck's constant.

Gold|Molecule|Gold junction geometry

To minimize the effect of variations in electron transport due to the contacting modalities to the electrode using thiomethyl anchor groups, we considered two different molecule | electrode configurations, where the thiomethyl sulfur atoms contact a single gold atom of each electrode (type I) or each sulfur atom contacts two gold atoms per electrode (type II) as described by Daaoub et al.[20] In addition to traditional S…S contacted junctions we also considered the

possibility of contact by the pyridine nitrogen denoted by ‘N’ or by contact with the metal center denoted by ‘M’.

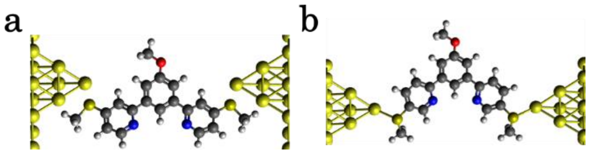

Figure S30. Schematic diagram of molecules **a**) $\mathbf{L^m}$H-I, **b**) $\mathbf{L^p}$H-I.

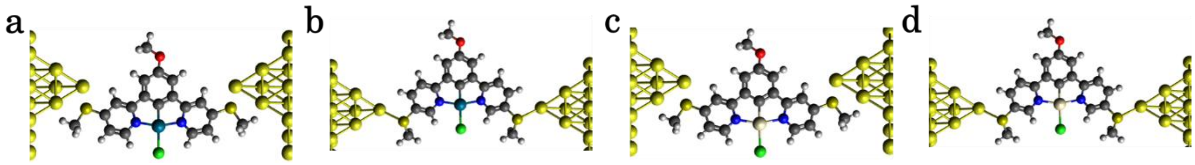

Figure S31. Schematic diagram of molecules **a**) $\mathbf{Pd^m}$-I, **b**) $\mathbf{Pd^p}$-I, **c**) $\mathbf{Pt^m}$-I and **d**) $\mathbf{Pt^p}$-I.

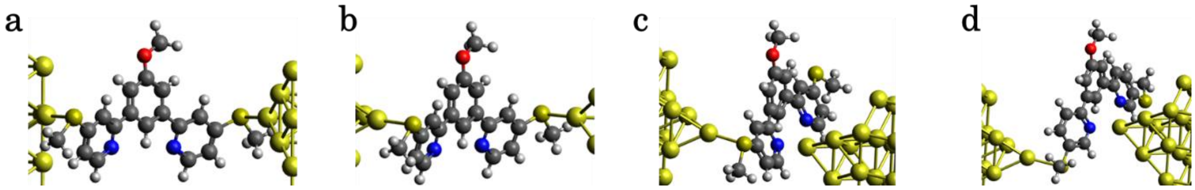

Figure S32. Schematic diagram of molecules **a**) $\mathbf{L^m}$H-II, **b**) $\mathbf{L^p}$H-II, **c**) $\mathbf{L^m}$H-N and **d**) $\mathbf{L^p}$H-N.

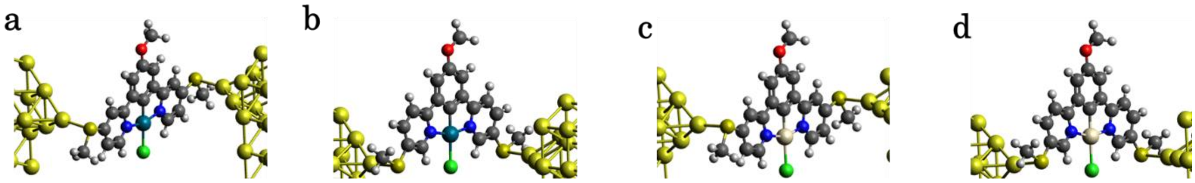

Figure S33. Schematic diagram of molecules **a**) $\mathbf{Pd^m}$-II, **b**) $\mathbf{Pd^p}$-II, **c**) $\mathbf{Pt^m}$-II and **d**) $\mathbf{Pt^p}$-II.

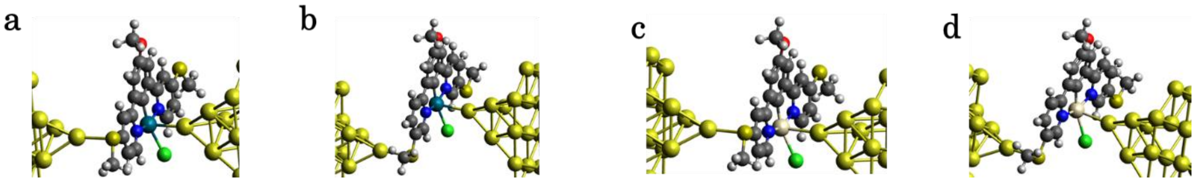

Figure S34. Schematic diagram of molecules **a**) $\mathbf{Pd^m}$-N, **b**) $\mathbf{Pd^p}$-N, **c**) $\mathbf{Pt^m}$-N and **d**) $\mathbf{Pt^p}$-N.

In addition to the possibility of forming a junction containing a single molecule between two electrodes we also considered the possibility of a molecular dimer forming in the junction. Here we consider the possible configurations previously explored in Figures S26 – S28.

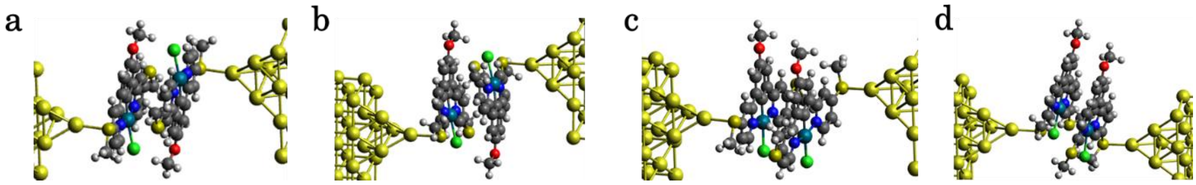

Figure S35. Schematic diagram of molecules **a**) **Pd**^{m}-sym, **b**) **Pd**^{p}-sym, **c**) **Pd**^{m}-asym and **d**) **Pd**^{p}-asym.

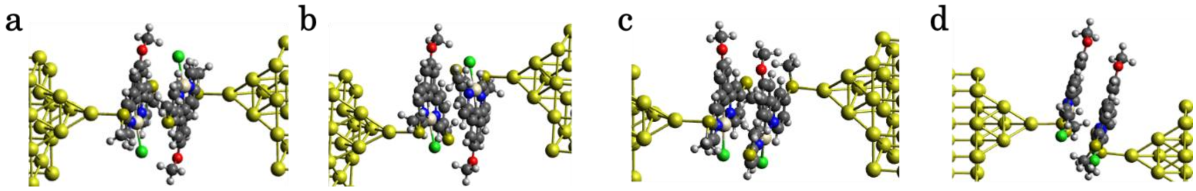

Figure S36. Schematic diagram of molecules **a**) **Pt**^{m}-sym, **b**) **Pt**^{p}-sym, **c**) **Pt**^{m}-asym and **d**) **Pt**^{p}-asym.

Transmission and Electrical Conductance

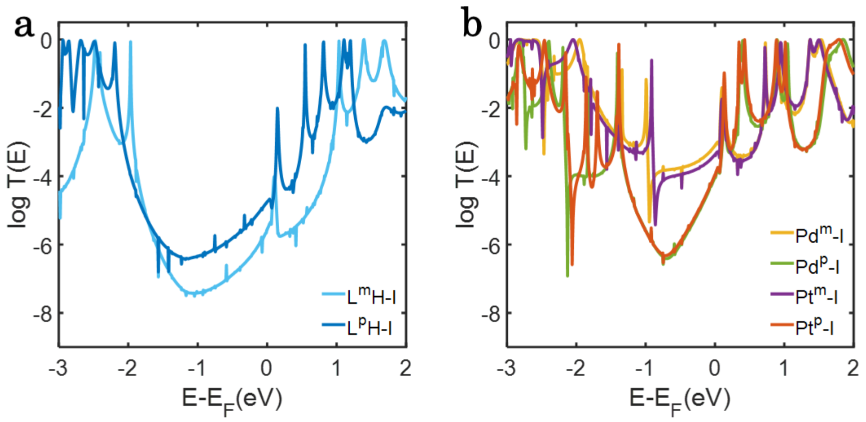

Figure S37. **a**) DFT electron transmission for the molecules **L^m**H, **L^p**H, **Pt^m**, **Pt^p**, **Pd^m**, **Pd^p**.

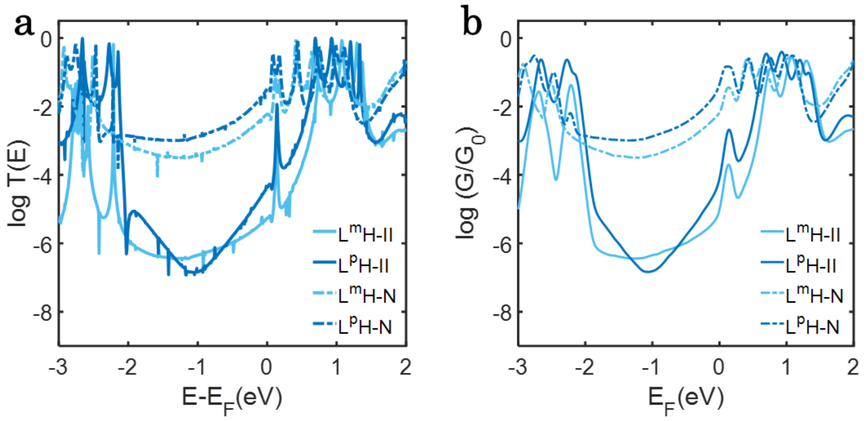

Figure S38. **a**) DFT electron transmission and **b**) conductance for the molecules **L^m**H, **L^p**H, **L^m**H-N and **L^p**H-N.

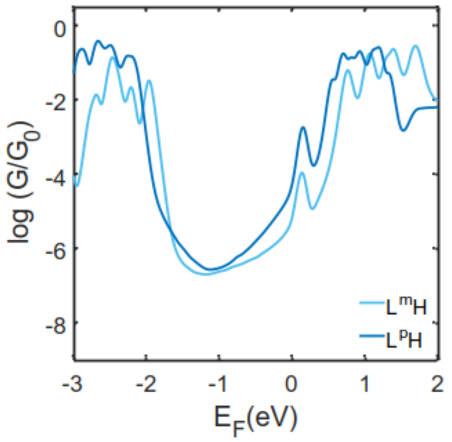

Figure S39. Unweighted average DFT conductance for the molecules **L^m**H, **L^p**H using two different configurations I, II to the electrodes.

Platinum and Palladium Complexes (Pt^{m}, Pt^{p}, Pd^{m}, Pd^{p})

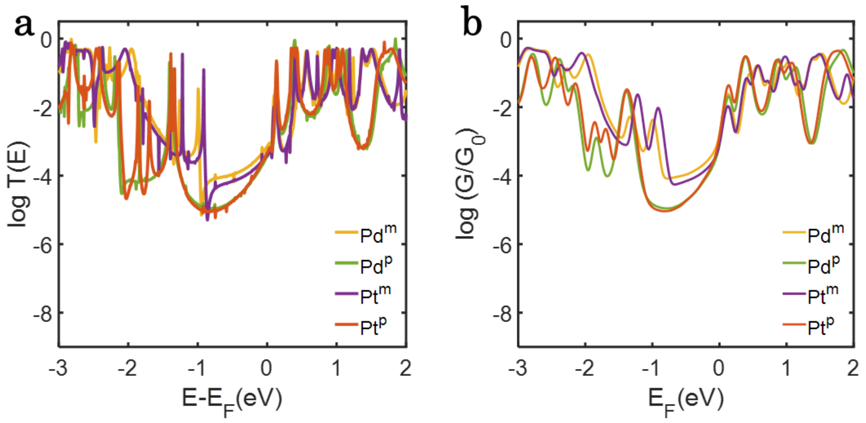

Figure S40. **a**) DFT electron transmission and **b**) conductance for the molecules **Pd^{m}**, **Pd^{p}**, **Pt^{m}** and **Pt^{p}**.

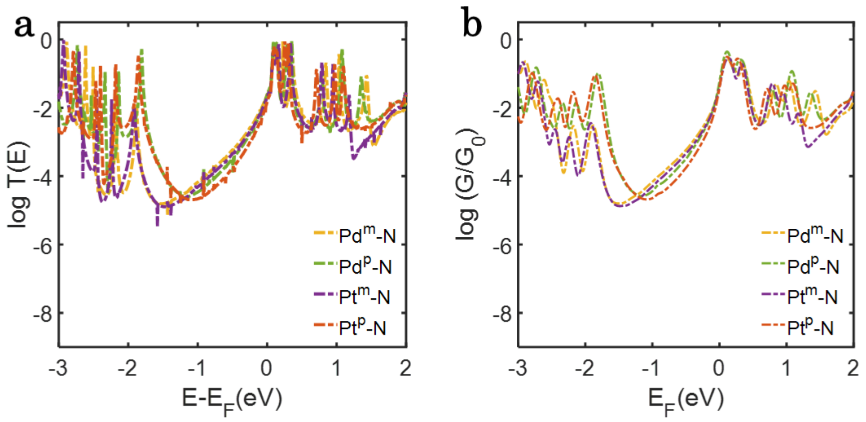

Figure S41. **a**) DFT electron transmission and **b**) conductance for the molecules **Pd^{m}**-N, **Pd^{p}**-N, **Pt^{m}**-N and **Pt^{p}**-N.

***Meta* vs. *Para* Isomers**

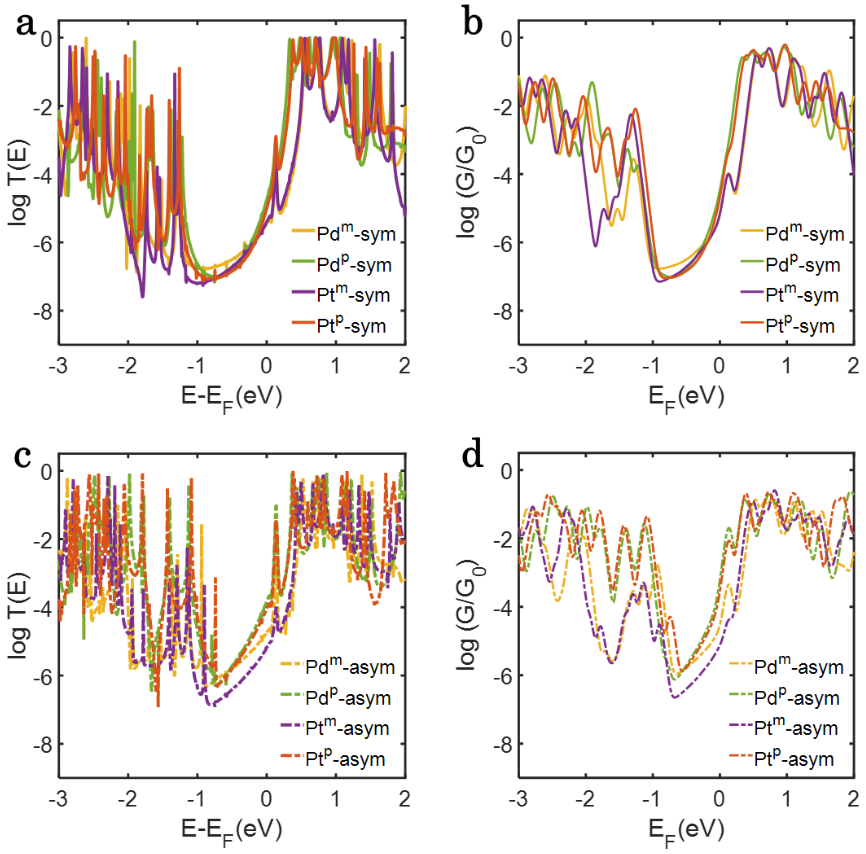

Figure S42. **a**) DFT electron transmission and **b**) conductance for the molecules **Pd^{m}**-sym, **Pd^{p}**-sym, **Pt^{m}**-sym and **Pt^{p}**-sym. **c**) DFT electron transmission and **d**) conductance for the molecules **Pd^{m}**-asym, **Pd^{p}**-asym, **Pt^{m}**-asym and **Pt^{p}**-asym.

Frontier orbital comparison

To offer insight into the conductance behavior of the compounds, we first examined the difference in orbital distribution. DFT calculations were performed on Gaussian09[21] using B3LYP; for the ligands (**L^{m}**H and **L^{p}**H) a 6-31G(d) basis set was employed for all the atoms, while for both the palladium (**Pd^{m}** and **Pd^{p}**) and the platinum (**Pt**^{m} and **Pt^{p}**) complexes a 6-31G(d)/LANL2DZ basis was used. To reduce computational complexity, the hexyl chains were approximated with methyl groups.

Both ligands showed LUMOs delocalized across the entire molecule, with the HOMOs localized to phenyl-pyridine units with limited contribution from the remaining pyridine (see Figure S42). Once coordinated to either Pd or Pt, the HOMO localized to the metal center and phenylate group, similarly to those systems reported by Sotoyama *et al.*,[22] while the LUMO remained delocalized over the ligand with little contribution from the metal center (see Figures S44 and S45).

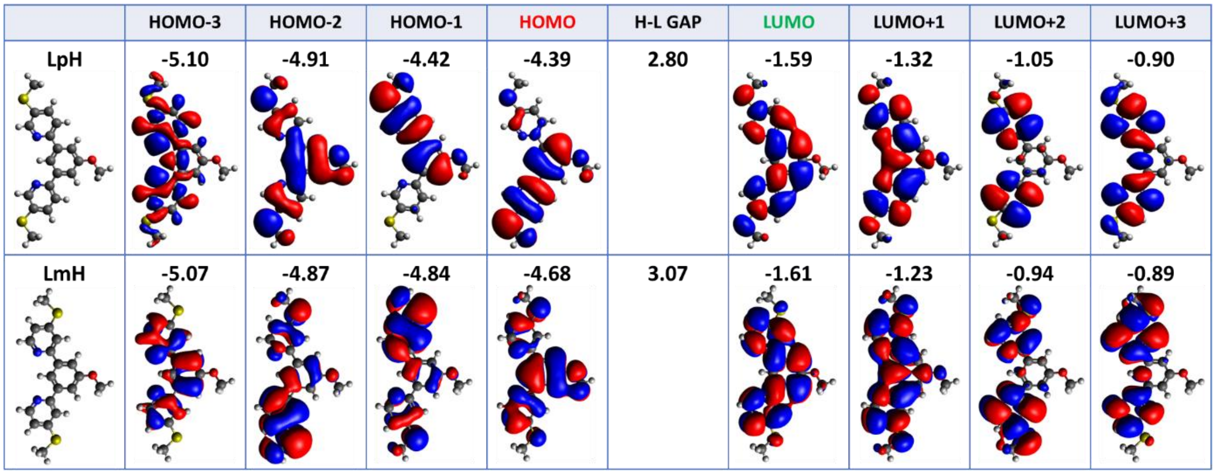

	HOMO-3	HOMO-2	HOMO-1	HOMO	H-L GAP	LUMO	LUMO+1	LUMO+2	LUMO+3
LpH	-5.10	-4.91	-4.42	-4.39	2.80	-1.59	-1.32	-1.05	-0.90
LmH	-5.07	-4.87	-4.84	-4.68	3.07	-1.61	-1.23	-0.94	-0.89

Figure S43. DFT wave function orbitals with corresponding energy values of **L**^{p}H and **L**^{m}H,

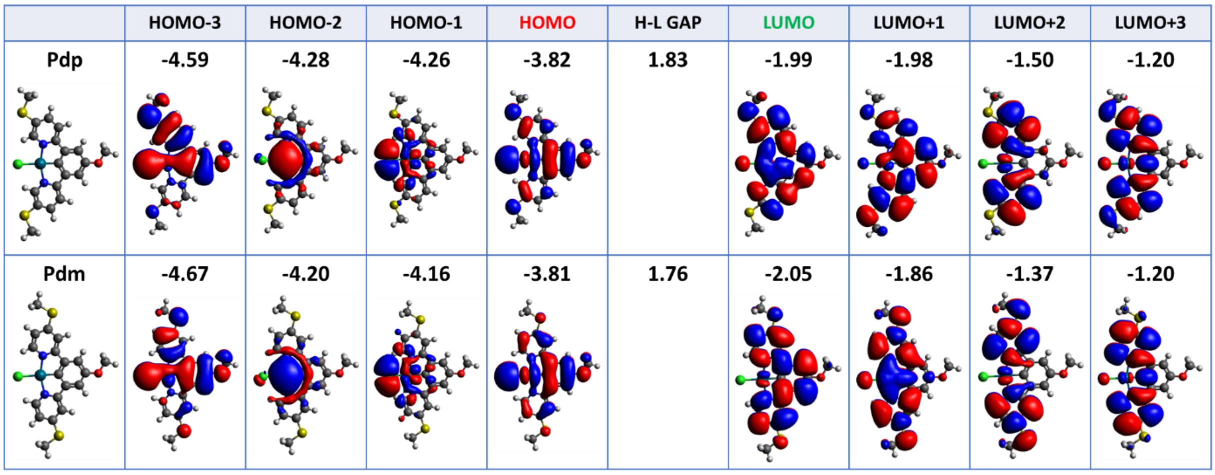

	HOMO-3	HOMO-2	HOMO-1	HOMO	H-L GAP	LUMO	LUMO+1	LUMO+2	LUMO+3
Pdp	-4.59	-4.28	-4.26	-3.82	1.83	-1.99	-1.98	-1.50	-1.20
Pdm	-4.67	-4.20	-4.16	-3.81	1.76	-2.05	-1.86	-1.37	-1.20

Figure S44. DFT wave function orbitals with corresponding energy values of **Pd**^{p} and **Pd**^{m}.

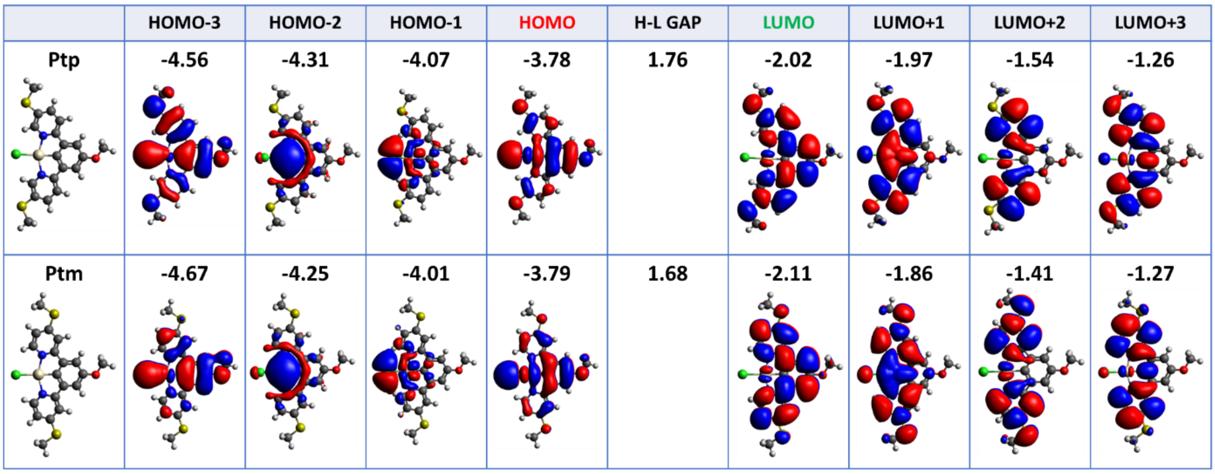

Figure S45. DFT wave function orbitals with corresponding energy values of **Pt^{p}** and **Pt^{m}**.

Table S7. HOMO-LUMO energy gap for all the measured compounds

**Compound**	**ΔE(H-L) (eV)**	
	**DFT**	**Measured[a]**
**L^{m}H**	3.07	3.58
**L^{p}H**	2.80	3.52
**Pd^{m}**	1.76	2.91
**Pd^{p}**	1.83	2.84
**Pt^{m}**	1.68	2.62
**Pt^{p}**	1.76	2.58

[a] Values based on electronic absorbance measurements (Figure S16 and Table S3).

References

(1) Li, J.; Lynch, M. P.; DeMello, K. L.; Sakya, S. M.; Cheng, H.; Rafka, R. J.; Bronk, B. S.; Jaynes, B. H.; Kilroy, C.; Mann, D. W.; et al. In vitro and in vivo profile of 2-(3-di-fluoromethyl-5-phenylpyrazol-1-yl)-5-methanesulfonylpyridine, a potent, selective, and orally active canine COX-2 inhibitor. *Bioorganic & Medicinal Chemistry* **2005**, *13* (5), 1805-1809. DOI: 10.1016/j.bmc.2004.11.048.

(2) Bastante, P.; Davidson, R. J.; Al Malki, W.; Salthouse, R. J.; Cea, P.; Martin, S.; Batsanov, A. S.; Lambert, C. J.; Bryce, M. R.; Agrait, N. The Conductance and Thermopower Behavior of Pendent *Trans*-Coordinated Palladium(II) Complexes in Single-Molecule Junctions. *ACS Omega* **2024**, *9*, 38303-38312. DOI: 10.1021/acsomega.4c06475.

(3) Krause, L.; Herbst-Irmer, R.; Sheldrick, G. M.; Stalke, D. Comparison of silver and molybdenum microfocus X-ray sources for single-crystal structure determination. *Journal of Applied Crystallography* **2015**, *48* (1), 3-10. DOI: doi:10.1107/S1600576714022985.

(4) Sheldrick, G. SHELXT - Integrated space-group and crystal-structure determination. *Acta Crystallographica Section A* **2015**, *71* (1), 3-8. DOI: doi:10.1107/S2053273314026370.

(5) Sheldrick, G. Crystal structure refinement with SHELXL. *Acta Crystallographica Section C* **2015**, *71* (1), 3-8. DOI: doi:10.1107/S2053229614024218.

(6) Dolomanov, O. V.; Bourhis, L. J.; Gildea, R. J.; Howard, J. A. K.; Puschmann, H. OLEX2: a complete structure solution, refinement and analysis program. *Journal of Applied Crystallography* **2009**, *42* (2), 339-341. DOI: doi:10.1107/S0021889808042726.

(7) Davidson, R.; Hsu, Y.; Batchelor, T.; Yufit, D.; Beeby, A. The use of organolithium reagents for the synthesis of 4-aryl-2-phenylpyridines and their corresponding iridium(III) complexes. *Dalton Transactions* **2016**, *45* (28), 11496-11507. DOI: 10.1039/C6DT01461E.

(8) Melhuish, W. H. QUANTUM EFFICIENCIES OF FLUORESCENCE OF ORGANIC SUBSTANCES: EFFECT OF SOLVENT AND CONCENTRATION OF THE

FLUORESCENT SOLUTE1. *Journal of Physical Chemistry* **1961**, *65* (2), 229-235. DOI: 10.1021/j100820a009.

(9) Wang, Z.; Turner, E.; Mahoney, V.; Madakuni, S.; Groy, T.; Li, J. Facile Synthesis and Characterization of Phosphorescent Pt(N∧C∧N)X Complexes. *Inorganic Chemistry* **2010**, *49* (24), 11276-11286. DOI: 10.1021/ic100740e.

(10) Rausch, A. F.; Murphy, L.; Williams, J. A. G.; Yersin, H. Improving the Performance of Pt(II) Complexes for Blue Light Emission by Enhancing the Molecular Rigidity. *Inorganic Chemistry* **2012**, *51* (1), 312-319. DOI: 10.1021/ic201664v.

(11) Colombo, A.; Fiorini, F.; Septiadi, D.; Dragonetti, C.; Nisic, F.; Valore, A.; Roberto, D.; Mauro, M.; De Cola, L. Neutral N^C^N terdentate luminescent Pt(II) complexes: their synthesis, photophysical properties, and bio-imaging applications. *Dalton Transactions* **2015**, *44* (18), 8478-8487. DOI: 10.1039/C4DT03165B.

(12) Greczynski, G.; Hultman, L. X-ray photoelectron spectroscopy: Towards reliable binding energy referencing. *Progress in Materials Science* **2020**, *107*, 100591. DOI: 10.1016/j.pmatsci.2019.100591.

(13) Major, G. H.; Fairley, N.; Sherwood, P. M. A.; Linford, M. R.; Terry, J.; Fernandez, V.; Artyushkova, K. Practical guide for curve fitting in x-ray photoelectron spectroscopy. *Journal of Vacuum Science & Technology A* **2020**, *38* (6). DOI: 10.1116/6.0000377.

(14) Evangeli, C.; Gillemot, K.; Leary, E.; González, M. T.; Rubio-Bollinger, G.; Lambert, C. J.; Agraït, N. Engineering the Thermopower of C_{60} Molecular Junctions. *Nano Letters* **2013**, *13* (5), 2141-2145. DOI: 10.1021/nl400579g.

(15) Grace, I. M.; Olsen, G.; Hurtado-Gallego, J.; Rincón-García, L.; Rubio-Bollinger, G.; Bryce, M. R.; Agraït, N.; Lambert, C. J. Connectivity dependent thermopower of bridged biphenyl molecules in single-molecule junctions. *Nanoscale* **2020**, *12* (27), 14682-14688. DOI: 10.1039/D0NR04001K.

(16) Miao, R.; Xu, H.; Skripnik, M.; Cui, L.; Wang, K.; Pedersen, K. G. L.; Leijnse, M.; Pauly, F.; Wärnmark, K.; Meyhofer, E.; et al. Influence of Quantum Interference on the Thermoelectric Properties of Molecular Junctions. *Nano Letters* **2018**, *18* (9), 5666-5672. DOI: 10.1021/acs.nanolett.8b02207.

(17) Soler, J. M.; Artacho, E.; Gale, J. D.; Garcia, A.; Junquera, J.; Ordejon, P.; Sanchez-Portal, D. The SIESTA method for ab initio order-N materials simulation. *Journal of Physics: Condensed Matter* **2002**, *14* (11), 2745-2779. DOI: 10.1088/0953-8984/14/11/302.

(18) Ferrer, J.; Lambert, C. J.; Garcia-Suarez, V. M.; Manrique, D. Z.; Visontai, D.; Oroszlany, L.; Rodriguez-Ferradas, R.; Grace, I.; Bailey, S. W. D.; Gillemot, K.; et al. GOLLUM: a next-generation simulation tool for electron, thermal and spin transport. *New Journal of Physics* **2014**, *16*. DOI: 10.1088/1367-2630/16/9/093029.

(19) Sadeghi, H. Theory of electron, phonon and spin transport in nanoscale quantum devices. *Nanotechnology* **2018**, *29* (37), 373001. DOI: 10.1088/1361-6528/aace21.

(20) Daaoub, A.; Ornago, L.; Vogel, D.; Bastante, P.; Sangtarash, S.; Parmeggiani, M.; Kamer, J.; Agraït, N.; Mayor, M.; van der Zant, H.; et al. Engineering Transport Orbitals in Single-Molecule Junctions. *The Journal of Physical Chemistry Letters* **2022**, *13* (39), 9156-9164. DOI: 10.1021/acs.jpclett.2c01851.

(21) *Gaussian 09*; Gaussian, Inc: Wallingford CT, 2009.

(22) Sotoyama, W.; Satoh, T.; Sato, H.; Matsuura, A.; Sawatari, N. Excited States of Phosphorescent Platinum(II) Complexes Containing N∧C∧N-Coordinating Tridentate Ligands: Spectroscopic Investigations and Time-Dependent Density Functional Theory Calculations. *The Journal of Physical Chemistry A* **2005**, *109* (43), 9760-9766. DOI: 10.1021/jp053366c.
